# Renqing Mangjue modulates chronic atrophic gastritis inflammation-cancer transition via the cGMP–PKG/PI3K–AKT pathway

**DOI:** 10.1186/s13020-026-01457-2

**Published:** 2026-07-14

**Authors:** Fengyu Huang, Peiping Chen, Daiyue Ding, Changyue Song, Xiangying Kong, Haiping Wang, Hua Zhou, Tao Su, Na Lin, Xiaohui Su

**Affiliations:** 1 Institute of Chinese Materia Medica, China Academy of Chinese Medical Sciences, Beijing, 100700 China; 2 State Key Laboratory of Traditional Chinese Medicine Syndrome, Guangdong Provincial Academy of Chinese Medical Sciences, Guangdong Provincial Hospital of Chinese Medicine, Guangzhou, 510006 China; 3 Guangzhou University of Chinese Medicine, Guangzhou, 510006 China; 4 Arura Tibetan Medicine Co. Ltd, Xining, 810003 China

**Keywords:** Chronic atrophic gastritis, Gastric cancer, Inflammation-cancer transition, Renqing Mangjue, cGMP–PKG/PI3K–AKT signaling pathway, Inflammatory cytokines

## Abstract

**Background:**

Chronic atrophic gastritis (CAG) is a critical stage in the progression from inflammation to cancer and is closely associated with an increased risk of gastric cancer (GC). Renqing Mangjue (RQMJ), a traditional Tibetan herbal remedy, has shown diverse pharmacological properties, including regulation of gastrointestinal function and anti-inflammatory effects. However, the specific effects and mechanisms by which RQMJ influences the transition from inflammation to cancer remain unclear.

**Objectives:**

This study aimed to evaluate the preventive and therapeutic effects of RQMJ on CAG and its intervention in the inflammation–cancer transition process, with a focus on exploring the underlying molecular mechanisms.

**Methods:**

RQMJ was chemically characterized by UPLC-Q-TOF–MS/MS and HPLC. A rat model of CAG and its inflammation-cancer transition was established and treated with different doses of RQMJ. The therapeutic effects of RQMJ were evaluated by gross gastric mucosal observation, ELISA, histopathological staining, immunohistochemistry, and Western blot analysis. In vitro, the effects of RQMJ on inflammation and migration of the gastric precancerous cell model (MC cells) were assessed using ELISA, wound-healing, Transwell migration, and Western blot assays. RNA sequencing and molecular biology techniques were then employed to explore the underlying mechanisms, and key targets were further validated in both in vivo and in vitro experiments. Finally, C-type natriuretic peptide (CNP) was used to verify the involvement of the identified signaling pathway.

**Results:**

A total of 2780 chemical constituents were identified in RQMJ. A dynamic rat model of CAG and its progression to GC was established. During CAG, persistent inflammation and aberrant PI3K–AKT activation formed a positive-feedback loop, driving FAK overexpression and epithelial–mesenchymal transition (EMT)-related changes, which remained active in GC despite partial reduction of inflammation. RQMJ markedly alleviated gastric mucosal injury, suppressed intestinal metaplasia, and improved gastrointestinal function. It also reduced pro-inflammatory mediators (TNF-α, IL-1β, CRP), increased IL-10, and downregulated tumor-related markers (Ki-67, Vimentin, CA19-9). In vitro, RQMJ inhibited MC cell proliferation and migration, suppressed inflammatory and EMT-related proteins. Mechanistically, RQMJ activated the cGMP–PKG pathway and inhibited the PI3K–AKT signaling pathway, thereby disrupting inflammatory amplification. This further downregulated extracellular matrix (ECM)–receptor interaction molecules (TGF-β, FAK, Itga2), ultimately blocking the inflammation–cancer transition.

**Conclusion:**

RQMJ may activate the cGMP–PKG signaling pathway while suppressing aberrant PI3K–AKT activation, thereby reducing inflammatory cytokine levels and inhibiting ECM remodeling and the EMT-associated process, and thus indirectly interrupting the inflammation**-**cancer cascade. These findings highlight the potential pharmacological value and therapeutic prospects of RQMJ in inflammation-driven gastric diseases.

**Graphical Abstract:**

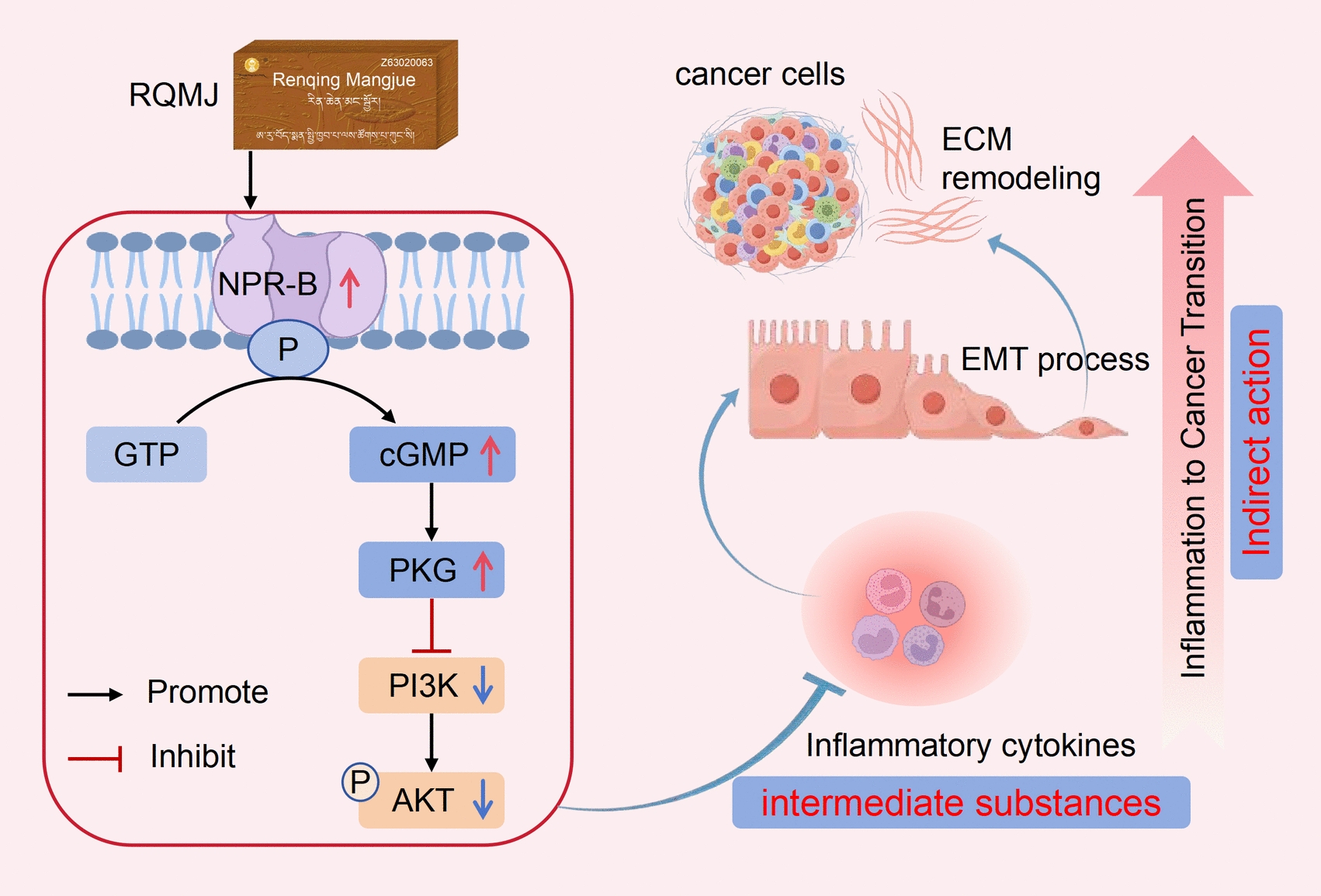

**Supplementary Information:**

The online version contains supplementary material available at 10.1186/s13020-026-01457-2.

## Introduction

Gastric cancer (GC) is the fifth most commonly diagnosed malignancy globally and ranks as the third leading cause of cancer-related mortality [1, 2]. The development of GC is a complex, multistep process involving various genetic, environmental, and inflammatory factors, contributing to its high invasiveness and metastatic potential [3]. As such, early detection and intervention are critical to reducing the global burden of GC. Increasing evidence indicates that GC develops through a progressive pathological process, beginning with chronic inflammation. In 1992, Correa proposed the “cascade model” of gastric carcinogenesis, which outlines a sequential progression from chronic gastritis to atrophic gastritis (CAG), intestinal metaplasia, and dysplasia, all of which represent recognized stages of precancerous gastric lesions [4]. Among these stages, CAG is a significant risk factor for GC and serves as a critical point in the inflammation-cancer transition. Epidemiological studies have confirmed a strong association between CAG and GC risk, with an increasing rate of progression over time [5]. Consequently, interventions targeting the progression from CAG to GC hold great promise in reducing the incidence of GC. However, effective therapeutic strategies for this pivotal stage remain limited [6].

Tibetan medicine, one of the four major traditional medical systems in the world, has a history spanning over three millennia. Its theoretical framework is built upon the “three humors theory”, which includes *rLung* (Wind), *MKhris pa* (Bile), and *Bad gen* (Phlegm)—concepts central to the understanding and treatment of digestive diseases [7, 8]. Chronic gastritis, recognized as a treatable condition in Tibetan medicine, can be managed through therapies that slow disease progression, prevent worsening, and even reverse pathological changes, thus potentially safeguarding against inflammation-driven carcinogenesis [9]. Renqing Mangjue (RQMJ), a rare Tibetan medicine with national recognition, is derived from classical Tibetan medical texts such as the *Four Medical Tantras* [10]. Its main ingredients, including *Terminalia chebula* Retz. (Maohezi), *Syzygium jambos* (L.) Alston (Putao), *Crocus sativus* L. (Zanghonghua), *Bovis Calculus* (Niuhuang), and *Moschus* (Shexiang), possess properties that clear heat, detoxify, soothe the liver, nourish the stomach, and relieve toxins [11]. RQMJ is a traditional Tibetan medicine that has long been used in clinical practice for gastrointestinal disorders. However, the detailed mechanisms through which RQMJ exerts its effects in chronic gastritis, particularly in preventing the inflammation-cancer transition, remain inadequately understood and require further investigation.

Inflammation plays a central role in tumor development, influencing key processes such as initiation, promotion, malignant transformation, invasion, and metastasis [12, 13]. Pro-inflammatory cytokines, including tumor necrosis factor-alpha (TNF-α) and interleukin-1 beta (IL-1β), are elevated in chronic inflammatory conditions and can activate multiple oncogenic signaling pathways. For example, IL-1β activates the phosphoinositide 3-kinase (PI3K)–protein kinase B (AKT) and mitogen-activated protein kinase kinase (MEK)/extracellular signal-regulated kinase (ERK) pathways, leading to epithelial-mesenchymal transition (EMT), migration, and invasiveness. Similarly, TNF-α regulates the expression of E-cadherin and Vimentin through the PI3K–AKT and glycogen synthase kinase-3 beta (GSK-3β) pathways, contributing to EMT. Importantly, persistent activation of the PI3K–AKT signaling pathway can further amplify the production of pro-inflammatory cytokines through downstream effectors such as nuclear factor kappa-B (NF-κB), thereby establishing a mutually reinforcing positive feedback loop between inflammatory mediators and oncogenic pathways. This self-sustaining “inflammation–oncogenesis” circuit not only promotes aberrant cell proliferation, inhibits apoptosis, and enhances immune evasion, but also drives the progression from chronic inflammation to cancer. Therefore, regulating inflammatory cytokine levels may disrupt this positive feedback loop, indirectly restrain tumor progression, and mechanistically contribute to suppressing carcinogenesis [14]. Accordingly, targeting the inflammatory signaling networks that govern this transition represents a promising therapeutic strategy.

This study aimed to investigate the role of RQMJ in the critical inflammation-cancer transition in CAG. Using rat models of CAG and its dynamic progression to GC, together with an MNNG-induced gastric precancerous cell model (MC cells), we systematically evaluated the preventive and therapeutic potential of RQMJ in gastric inflammation–carcinogenesis. Transcriptomic analysis combined with molecular biological experiments was further performed to elucidate the role of RQMJ in the inflammation–cancer progression cascade and its regulatory effects on the cyclic guanosine monophosphate (cGMP)–cGMP-dependent protein kinase (PKG)/PI3K–AKT signaling pathways. In addition, C-type natriuretic peptide (CNP)-mediated pathway activation was used for mechanistic validation. This multi-dimensional integrative approach provides new evidence supporting the therapeutic potential of RQMJ and offers a theoretical basis for delaying or interrupting inflammation-driven malignant transformation of the gastric mucosa. Ultimately, RQMJ may represent a promising candidate for the prevention and treatment of GC.

## Materials and methods

### Chemicals and reagents

RQMJ Powder (Lot# 01231213) was purchased from Arura Tibetan Medicine Co., Ltd., China. Weifuchun Capsule (WFC, Z20090697) was obtained from Hangzhou Hu Qing Yu Tang Pharmaceutical Co., Ltd., China. N-methyl-N′-nitro-N-nitrosoguanidine (MNNG, Cat# M91260) was sourced from Shanghai Jizhi Biochemical Technology Co., Ltd., China. Ranitidine Hydrochloride Capsules (Cat# H44021173) were purchased from Guangdong Hengjian Pharmaceutical Co., Ltd., China. Medicated feed containing 0.05% ranitidine hydrochloride (Lot# 1209162400031039, Production License No. SCXK (Jin) 2020–0004) was acquired from Ke’ao Xieli (Tianjin) Feed Co., Ltd., China. 46% red star erguotou liquor (Cat# 6906785022364) was sourced from Beijing Red Star Co., Ltd., China. Hematoxylin and eosin (HE) staining kit (Cat# G1120) was purchased from Beijing Solarbio Science & Technology Co., Ltd. (Beijing, China). The alcian blue–periodic acid–Schiff (AB-PAS) staining kit (Cat# BA4121) was obtained from Zhuhai Beso Biotechnology Co., Ltd., China. Rat enzyme-linked immunosorbent assay (ELISA) kits for C-reactive protein (CRP, Cat# E-EL-R0506), TNF-α (Cat# E-EL-R2856), IL-1β (Cat# E-EL-R0012), interleukin-10 (IL-10, Cat# E-EL-R0016), gastrin (GAS, Cat# E-EL-R0472), motilin (MTL, Cat# E-EL-R0639), and pepsin activity (Cat# E-EL-K844-M) were purchased from Elabscience Biotechnology Inc. (Wuhan, China). The rat carbohydrate antigen 19–9 ELISA kit (CA19-9, Cat# ml106468) was sourced from Shanghai Enzyme-linked Biotechnology Co., Ltd., China. Primary antibodies used included horseradish peroxidase (HRP)-conjugated goat anti-mouse IgG and HRP-conjugated goat anti-rabbit IgG (Cat# 7076, Cat# 7074, Cell Signaling Technology, Danvers, MA, USA); Ki-67 (Cat# ab15580) and matrix metalloproteinase-9 (MMP-9, Cat# ab76003, Abcam, UK); beta-actin (β-actin, Cat# 81115–1-RR), natriuretic peptide receptor-B (NPR-B, Cat# 55113–1-AP), and cyclic guanosine monophosphate (cGMP)-dependent protein kinase (PKG, Cat# 55138–1-AP, Proteintech, USA). Additional antibodies included PI3K, phospho-AKT (p-AKT), and AKT (Cat# C73F8, Cat# Ser473, Cat# C67E7, Cell Signaling Technology, USA); TNF-α (Cat# sc52746), and Vimentin (Cat# sc6260, Santa Cruz Biotechnology, USA); IL-1β (Cat# NB600-633, Novus, Centennial, CO, USA); transforming growth factor-β (TGF-β, Cat# F1360), focal adhesion kinase (FAK, Cat# F3256), and integrin alpha-2 (Itga2, Cat# F1694) were purchased from Selleck (USA); tribromoethanol (M2920) was from Nanjing Aibei Biotechnology Co.,Ltd., China. Human gastric mucosal epithelial cells (GES-1) were purchased from Meilun Biotechnology Co., Ltd. (Cat# CL0187, Dalian, China). Transwell 12-well inserts were obtained from Corning (Cat# 3422, USA). Crystal violet was purchased from Solarbio (Cat# C8470, China). C-type natriuretic peptide (32–53) (CNP, Cat# 4095840) was obtained from Bachem AG (Bubendorf, Switzerland). Dulbecco's Modified Eagle Medium (DMEM, Cat# C11995500BT, Gibco, USA). Fetal bovine serum (FBS, Cat# 16000–044, Gibco, USA).

### Medicinal materials of RQMJ

The formulation of RQMJ is a proprietary Tibetan medicinal product manufactured by Arura Tibetan Medicine Co., Ltd. (Qinghai Province, China) in accordance with Good Manufacturing Practice (GMP) standards. The principal herbal ingredients are listed in Table 1. The exact composition ratios are proprietary to the manufacturer. Nevertheless, the formulation has been standardized and quality-controlled to ensure consistency across batches.

**Table 1:** Medicinal materials of RQMJ

Plant name	Chinese name	English name
*Terminalia chebula* Retz	Maohezi	myrobalan
*Syzygium jambos* (L.) Alston	Putao	Malabar plum
*Crocus sativus* L	Xihonghua	Saffron
*Strychnos nux-vomica* L	Maqianzi	Nux Vomica
*Aquilaria sinensis* (Lour.) Spreng	Chenxiang	agilawood
*Tinospora sinensis* (Lour.) Merr	kuanjinteng	Chinese tinospora
*Santalum album* L	Baitanxiang	Sandalwood
*Pterocarpus santalinus* L.f	Zitan	Red sandalwood
*Swertia mussotii* Franch	Zangyinchen	Tibetan Swertia
*Piper longum* L	Biba	Long peppe
*Herpetospermum pedunculosum* (Ser.) C.B.Clarke	Bolenɡgua	Herpetospermum seed
*Holarrhena antidysenterica* Wall. ex A. DC	Zhixiemu	Holarrhena bark
*Inula racemosa* Hook.f	Zangmuxiang	Inula racemosa root
*Coriandrum sativum* L	Yansuiguo	Coriander
*Lagotis brevituba* Maxim	Duanguantuercao	Lagotis herb
*Boswellia sacra* Flück	Ruxiang	Frankincense
*Senna obtusifolia* (L.) H.S.Irwin & Barneby	Juemingzi	Sicklepod seed
*Abelmoschus moschatus* Medik	Huangkui	Musk mallow seed
*Uncaria rhynchophylla* (Miq.) Miq	Gouteng	Uncaria hook
*Styrax benzoin* Dryand	Anxixiang	Benzoin resin
*Brassica rapa* L	Manjing	Field mustard
*Braya humilis* (C.A.Mey.) B.L.Rob	Yinguojie	Braya herb
*Cyrtomium fortunei* J.Sm	Guanzhong	Fortune’s holly fern
*Pyrrosia lingua* (Thunb.) Farw	Shiwei	Tongue fern
*Corallodiscus lanuginosus* (Wall. ex R.Br.) B.L.Burtt	Shanhujutai	Coral bell
*Syzygium aromaticum* (L.) Merr. & L.M.Perry	Dingxiang	Clove
*Lanxangia tsao-ko* (Crevost & Lemarié) M.F.Newman & Škorničk	Caoguo	Black cardamom
*Bambusa textilis* McClure	Qingpizhu	Bamboo fiber
*Myristica fragrans* Houtt	Roudoukou	Nutmeg
*Malva verticillata* L	Dongkui	Chinese mallow
*Lygodium japonicum* (Thunb.) Sw	Haijinsha	Japanese climbing fern
*Butea monosperma* (Lam.) Kuntze	Zikuang	Butea flower
*Embelia laeta* (L.) Mez	Suantengzi	Embelia fruit
*Lagotis brachystachya* Maxim	Duansuituercao	Lagotis herb
*Areca catechu* L	Binglang	Areca nut
*Corydalis yanhusuo* (Y.H.Chou & Chun C.Hsu) W.T.Wang ex Z.Y.Su & C.Y.Wu	Yanhusuo	Corydalis tuber
*Phyllanthus emblica* L	Yuganzi	Indian gooseberry
*Aconitum tanguticum* (Maxim.) Stapf	Bangyi	Tangut aconite root
*Dolomiaea costus* (Falc.) Kasana & A.K.Pandey	Yunmuxiang	Costus root
*Mangifera indica* L	Wangguo	Mango
*Guilandina bonduc* L	Datuoyeyunshi	Bonduc nut
*Alpinia hainanensis* K.Schum	Caokou	Hainan galangal
*Piper cubeba* L.f	Bichengqie	Cubeb
*Phytolacca acinosa* Roxb	Shanglu	Indian pokeweed
*Aconitum pulchellum* Hand.-Mazz	Meiliwutou	Aconitum root
*Curcuma longa* L	Jianghuang	Turmeric

### Chemical profiling of RQMJ

The chemical constituents of RQMJ were analyzed using an ultra-performance liquid chromatography coupled with tandem mass spectrometry (UPLC–MS/MS) system. Detailed information on the analytical conditions, including instrument configuration and gradient program, is presented in Supplementary M2. Data acquisition and processing were conducted using Analyst software version 1.6.3 (AB Sciex).

### Animals

Male SPF-grade Wistar rats (100–120 g) were purchased from SPF (Beijing) Biotechnology Co., Ltd. (License No. SCXK (Beijing) 2024–0001). All experimental protocols were approved by the Animal Ethics Committee of the Institute of Basic Theory, China Academy of Chinese Medical Sciences (Approval No. IBTCMCACMSZI-2403–13), and all procedures adhered to the guidelines outlined in the National Institutes of Health (NIH) Guidelines for the Care and Use of Laboratory Animals.

### Establishment of rat models of CAG and inflammation–cancer transition

The rat models of CAG and inflammation–cancer transition were established using a multifactorial approach, as described in previous studies [15]. A total of 100 Wistar rats were used in this study and allocated to the Control group (*n* = 6) and experimental groups (*n* = 8 per group). Rats were given continuous access to 120 μg/mL MNNG in their drinking water, along with daily feeding of standard chow containing 0.05% ranitidine. Additionally, rats were subjected to a 16-h fasting period once weekly and intragastrically administered 1 mL of 46% ethanol per 200 g body weight on the fasting day. The Control group received standard chow and water ad libitum without any additional treatment. From week 16 to week 34, two rats from the model group were randomly selected every two weeks for histopathological analysis. These animals were used for sequential pathological evaluation and were not included in the final mechanistic analyses. During the long-term modeling process, an additional 8 rats were lost due to complications associated with repeated gavage and ethanol administration. Therefore, 6 rats per group successfully completed the full experimental protocol and were included in the final analyses.

### Grouping and administration

After a 7-day acclimatization period, the Wistar rats were randomly assigned to two experimental phases. In the first phase, the preventive and therapeutic effects of RQMJ on CAG were evaluated. The dose for the RQMJ medium-dose group (RQMJ-M) was determined based on the commonly used clinical dose in humans and converted across species using the body surface area normalization method, resulting in 90 mg/kg. Animals were divided into six groups: Control, CAG Model, Positive Control (WFC, 44 mg/kg), and three RQMJ treatment groups, receiving low (RQMJ-L, 45 mg/kg), medium (RQMJ-M, 90 mg/kg, clinically equivalent dose), and high (RQMJ-H, 180 mg/kg) doses, respectively. Treatment continued until week 18, with the endpoint defined by the emergence of typical CAG pathological features in the model group. In the second phase, the effect of RQMJ on the progression from inflammation to cancer in CAG was investigated. The animals were again divided into six groups: Control, GC Model, Positive Control (WFC, 44 mg/kg), and three RQMJ treatment groups, receiving low (RQMJ-L, 45 mg/kg), medium (RQMJ-M, 90 mg/kg), and high (RQMJ-H, 180 mg/kg) doses. From week 18 onwards, continuous treatment was maintained until week 34, with the emergence of typical GC pathological features in the model group rats serving as the endpoint for this phase.

### Specimen collection and processing

The general activity and other characteristic changes of the rats were observed daily, and body weight was recorded weekly. At designated sampling time points, the pathological stage of each rat, either CAG or GC, was assessed to guide sample collection. After anesthesia with tribromoethanol, blood samples were collected via the abdominal aorta. The entire stomach was then excised, incised along the greater curvature, and its contents were removed. The gastric lumen was thoroughly rinsed with normal saline, spread flat for photographic documentation, and subjected to gross morphological examination.

### Gastric histopathological evaluation

Gastric tissue from the antrum to the pylorus were collected and fixed in 4% paraformaldehyde for histopathological analysis. The tissues were then embedded and sectioned to a thickness of 5 μm, followed by HE staining. Pathological changes in the gastric tissue, including inflammatory cell infiltration and morphological alterations, were examined under an optical microscope.

### Immunohistochemical evaluation

After dewaxing and rehydration, the remaining tissue sections were rinsed with PBS and treated with 2–3 drops of a composite digestion and antigen retrieval solution for 20 min. Permeabilization was performed using 0.1% Triton X-100 for 15 min. To quench endogenous peroxidase activity, the sections were incubated with 3% hydrogen peroxide in methanol for 10 min. Nonspecific binding was then blocked by incubating the sections with 5% bovine serum albumin (BSA) at room temperature for 1 h. Primary antibodies targeting NPR-B (1:50), PKG (1:50), PI3K (1:100), p-AKT (1:100), Ki-67 (1:100), Vimentin (1:100), TNF-α (1:100), IL-1β (1:100), CD68 (1:50) and CD4 (1:50) were applied, and the sections were incubated overnight at 4 °C. After warming the sections to 37 °C for 30 min, secondary antibodies were added and incubated at 37 °C for 1 h, followed by washing in PBST. Visualization was achieved using diaminobenzidine (DAB) chromogenic staining, and nuclei were counterstained with hematoxylin. The sections were rinsed with tap water, dehydrated, cleared, and mounted. Images were captured using an optical microscope.

### AB-PAS staining

Paraffin-embedded gastric tissue sections were deparaffinized and rehydrated, then stained with Alcian Blue solution (pH 2.5) for 10–20 min. After a brief rinse, excess water was blotted, and the sections were oxidized with an iodine solution for 10 min, followed by rinsing with distilled water. The sections were then stained with Schiff reagent for 10–15 min and washed with running water. Nuclear staining was performed using hematoxylin, followed by rinsing with running water to achieve a blue coloration. The sections were then dehydrated, cleared, and mounted with neutral balsam. Finally, images were captured using an optical microscope. Intestinal metaplasia was qualitatively evaluated based on the presence, abundance, and distribution of PAS-positive goblet cells within the gastric mucosal glands. Goblet cells were identified by their characteristic goblet-like morphology and strong PAS-positive magenta staining in the cytoplasm, reflecting mucin accumulation. An increased number and wider distribution of PAS-positive goblet cells were considered indicative of more severe intestinal metaplasia.

### ELISA assay

Blood was collected from the abdominal aorta and allowed to stand at room temperature for 2 h, followed by centrifugation at 3000 rpm for 15 min. The supernatant was collected for subsequent analysis. GAS, MTL, TNF-α, IL-10, CA19-9 and cGMP levels in serum were measured using ELISA according to the manufacturer’s instructions. For gastric tissue collection, the tissue was rinsed with pre-cooled PBS, weighed, minced, and ground into powder in liquid nitrogen. A protease inhibitor (100:1) was added, and the tissue was lysed on ice for 1 h. The lysate was then centrifuged at 5000 × g for 5–10 min at 4 °C, and the supernatant was collected for further analysis. Levels of CRP, IL-1β, and pepsin activity in gastric tissue were determined using ELISA following the manufacturer’s protocol. For in vitro experiments, cGMP levels in cell culture supernatants were also measured using ELISA following the manufacturer’ s instructions.

### Protein extraction and western blot

Radioimmunoprecipitation assay (RIPA) buffer containing protease inhibitors was added to gastric tissue powder or cells, followed by lysis on ice for 60 min. The lysate was then centrifuged at 12,000 rpm for 5 min at 4 °C, and the supernatant was collected as total protein. Protein concentration was determined using the Coomassie brilliant blue assay. Protein samples were separated by polyacrylamide gel electrophoresis (PAGE) and transferred to polyvinylidene fluoride (PVDF) membranes using the wet transfer method. Membranes were blocked with 5% BSA and incubated overnight at 4 °C with primary antibodies against NPR-B (1:400), PKG (1:400), PI3K (1:1000), AKT (1:1000), p-AKT (1:1000), TNF-α (1:1000), IL-1β (1:1000), Vimentin (1:1000), TGF-β (1:1000), FAK (1:1000), Itga2 (1:1000), and β-actin (1:1000). After washing, the membranes were incubated with appropriate secondary antibodies (anti-mouse or anti-rabbit, 1:5000) for 2 h at room temperature on a shaker. Protein bands were visualized using an enhanced chemiluminescent (ECL) substrate and imaged with a gel documentation system.

### Transcriptome sequencing and data analysis

Total RNA was extracted and subjected to RNA sequencing (RNA-seq). Principal component analysis (PCA) was performed to assess the overall variability and clustering patterns of gene expression profiles across the different experimental groups. Differential gene expression (DGE) analysis was conducted using the criteria of |log₂ Fold Change|≥ 0.7 and *P*-value < 0.05. Volcano plots were generated using GraphPad Prism 10. Hierarchical clustering was visualized using the Weishengxin platform [16]. Core target genes potentially involved in the RQMJ-mediated intervention in the CAG inflammation–cancer transition were imported into the Database for Annotation, Visualization, and Integrated Discovery (DAVID) database for Kyoto Encyclopedia of Genes and Genomes (KEGG) enrichment analysis [17]. The top 10 enriched terms were visualized using the Weishengxin platform.

### Cell culture

GES-1 cells were cultured in DMEM supplemented with 10% fetal bovine serum, 100 U/L penicillin, and 100 mg/L streptomycin at 37 °C in a humidified incubator with 5% CO₂. When cell confluence reached approximately 80%, cells were trypsinized and passaged, and cells at passages 3–4 were used for subsequent experiments. Normal GES-1 cells were exposed to 20 μmol/L MNNG (as previously optimized by our research group) for 24 h in the dark to induce morphological changes. Cellular morphology was observed and recorded under an inverted microscope. The criteria for successful model establishment were defined according to previous reports [18]. Cells meeting these criteria were defined as the gastric precancerous cell model (MC cells).

Based on preliminary dose-screening results, to evaluate the effects of different treatments on MC cell proliferation, the following groups were established: Control group, Model group, positive control group (WFC, 200 μg/mL), RQMJ-L group (3 μg/mL), RQMJ-M group (10 μg/mL), and RQMJ-H group (30 μg/mL). Cells in each group were seeded into plates, with three replicate wells per group.

To further investigate the relationship between RQMJ, CNP, and the cGMP–PKG signaling pathway, the following groups were established: Control group, Model group, RQMJ-H group (RQMJ), CNP group (100 nM), and RQMJ + CNP combined treatment group. Cells in each group were seeded into plates, with three replicate wells per group.

### Scratch wound-healing assay

GES-1 and MC cell suspensions were prepared and seeded into 6-well plates at a density of 3 × 10^5^ cells per well. After cells reached confluence, a cross-shaped scratch was created in the central area of each well. The medium was then removed, and cells were washed three times with PBS. Serum-free medium containing RQMJ was added, and images were immediately captured at 0 h. Cells were subsequently incubated at 37 °C in a humidified atmosphere with 5% CO₂, and images were taken again at 24 h. Wound areas were quantified using ImageJ software by measuring three fields per well. The cell migration rate was calculated using the following formula: migration rate (%) = [(scratch area at 0 h − scratch area at 24 h)/scratch area at 0 h] × 100%.

### Transwell migration assay

Cell migration was assessed using a 12-well Transwell chamber. GES-1 and MC cells were prepared as a single-cell suspension at a density of 5 × 10^4^ cells/mL. A total of 100 μL cell suspension mixed with serum-free medium containing RQMJ was added to the upper chamber, while the lower chamber was filled with medium supplemented with 15% fetal bovine serum as a chemoattractant. After 24 h of incubation, cells were washed with PBS, fixed for 20 min, and stained with 0.1% crystal violet for 20 min. Finally, migrated cells were observed under a 100 × microscope. Three random fields per chamber were selected for imaging and cell counting.

### Statistical analyses

The data are expressed as the mean ± standard error of the mean (SEM). Each experiment was performed independently at least three times. Statistical significance was determined using one-way analysis of variance (ANOVA) with GraphPad Prism 10 software. Differences were considered statistically significant at *P* < 0.05 and *P* < 0.01.

## Results

### Chemical profiling of RQMJ

To comprehensively characterize the chemical composition of RQMJ, UPLC–MS/MS analysis was performed under both positive and negative ionization modes. The corresponding base peak chromatograms are presented in Supplementary Fig. 1A–B. A total of 2,780 chemical constituents were identified, including representative compounds such as Yomogin, Plantainoside A, Turcamine, and Machilusin (Supplementary M3). Furthermore, all detected metabolites were categorized according to their structural classes, as summarized in Supplementary Fig. 1C. These constituents were classified into 13 classes. Among them, flavonoids accounted for the largest proportion (16.01%), followed by alkaloids (15.79%), terpenoids (15.76%), and others (15.76%). Phenolic acids also represented a considerable fraction (12.73%), while lipids accounted for 7.23% and lignans and coumarins for 5.50%. In contrast, organic acids (3.38%), amino acids and derivatives (2.66%), quinones (1.94%), tannins (1.83%), nucleotides and derivatives (1.22%), and steroids (0.18%) were present in relatively smaller proportions. These findings indicate that RQMJ possesses a complex and diverse chemical composition, suggesting potential multi-component synergistic regulation. Accordingly, a whole-formula intervention strategy was employed in the following experiments.

### Progressive histopathological and molecular changes in the rat model of inflammation–cancer transition in CAG

**Fig. 1:**
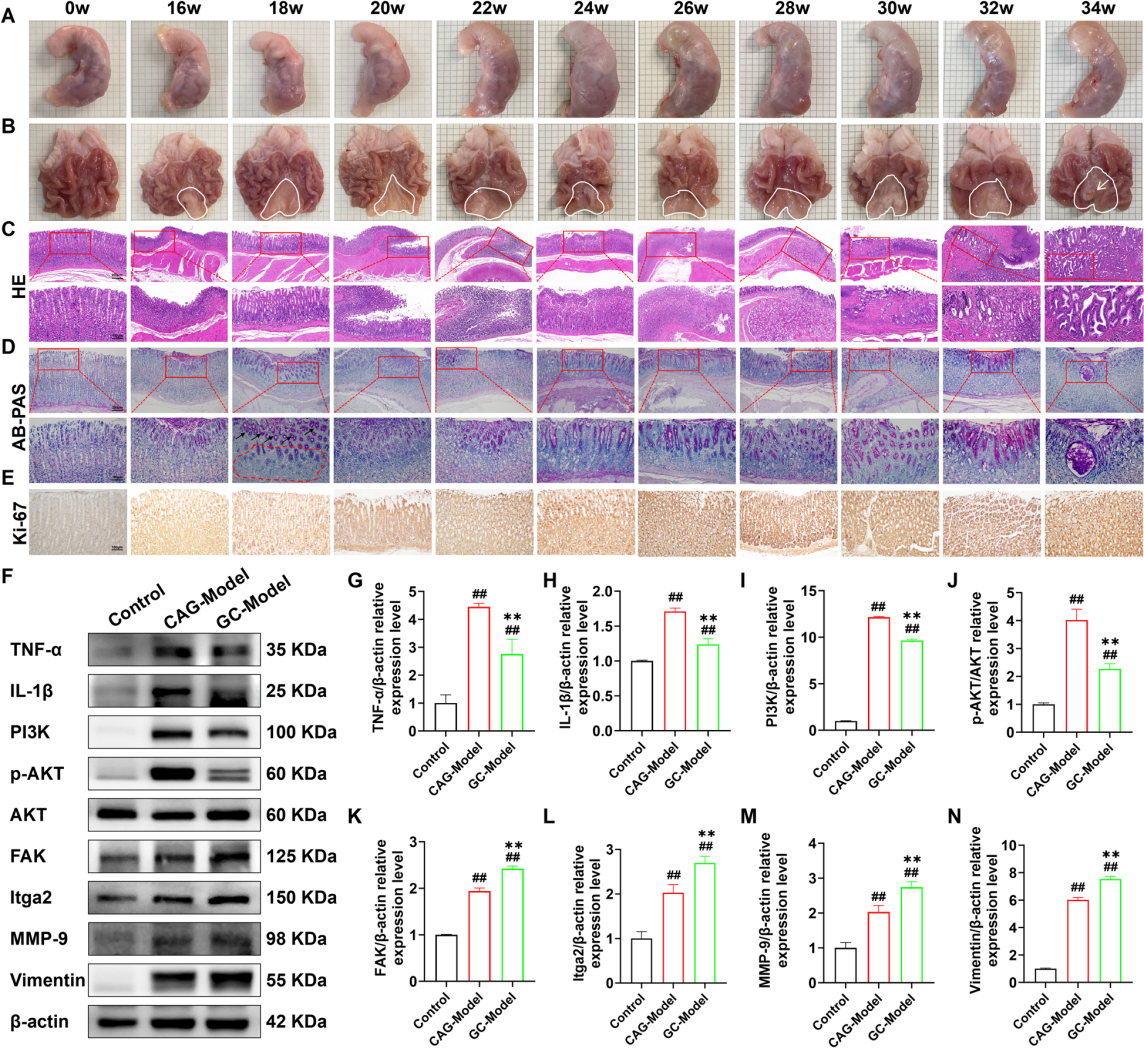
Dynamic Evolution of Gastric Lesions in the Rat Model of Inflammation–Cancer Transition. **A** Gross morphological images of the stomach at various time points during the model development, showing progressive changes in the gastric architecture, from normal to GC (*n* = 3). **B** Representative images of the gastric mucosa at the indicated time points, demonstrating the transition from normal mucosa to dysplasia and tumor formation (*n* = 3). **C** HE staining showing histological changes, including the thinning of gastric mucosa, inflammatory cell infiltration, and the development of dysplasia (*n* = 3, scale bar: 200 μm), with magnified views provided for the lesion sites (scale bar: 100 μm). **D** AB-PAS staining revealed the progression from normal gastric mucosa to intestinal metaplasia, with increased goblet cells (*n* = 3; scale bar: 200 μm), with magnified views provided for the lesion sites (scale bar: 100 μm). At week 18, intestinal metaplasia was observed, characterized by increased PAS-positive goblet cells (black arrows) and disorganized glandular architecture (dashed boxes). **E** Ki-67 staining demonstrating the proliferative activity in the gastric mucosa, with increased expression at later stages of disease (*n* = 3, scale bar: 100 μm). **F** Western blot analysis showing the expression of inflammatory and cancer-related proteins, including TNF-α, IL-1β, PI3K, p-AKT, AKT, FAK, Itga2, MMP-9, and Vimentin in Control, CAG Model, and GC Model rats (*n* = 3). **G-N** Quantification of protein expression levels for TNF-α, IL-1β, PI3K, FAK, Itga2, MMP-9, and Vimentin, with β-actin as the internal control (*n* = 3). Quantification of p-AKT expression normalized to total AKT (*n* = 3). The data are presented as mean ± SEM, with statistical significance denoted by ^#^*P* < 0.05, ^##^*P* < 0.01 compared to the Control group, and ^**^P < 0.01 compared to the CAG-Model group respectively (*n* = 3)

The progression of gastric mucosal lesions from CAG to GC in the rat model was characterized by significant morphological and molecular changes [19]. Gross morphological examination revealed that, by week 16, the gastric mucosa in model rats had thinned, with reduced rugae and early signs of inflammation. By week 34, distinct tumor masses were visible in the gastric fundus, accompanied by marked mucosal thinning and loss of rugae (Fig. 1A, B). Histological analysis using HE staining showed that at week 16, mild inflammatory cell infiltration was confined to the superficial mucosa. By week 18, the lesions progressed to intestinal metaplasia, evident by an increase in goblet cells and a disorganized glandular structure. At week 34, the tissue showed signs of dysplasia with disorganized glandular architecture, enlarged nuclei, and intraepithelial neoplasia, characteristic of the transition to GC (Fig. 1C). Further confirmation of the progression to intestinal metaplasia was provided by AB-PAS staining, which showed extensive blue staining corresponding to increased goblet cells in the mucosa at week 18, and pronounced proliferation of acid-secreting intestinal glands by week 34 (Fig. 1D). Additionally, Ki-67 staining revealed a significant increase in proliferative activity, with high expression in the mucosa and muscularis at week 34, reflecting the shift toward a more aggressive tumor phenotype (Fig. 1E). Western blot analysis was conducted to assess the expression levels of key inflammation- and cancer-related proteins. In both CAG and GC rats, proteins such as TNF-α, IL-1β, PI3K, p-AKT, FAK, Itga2, MMP-9, and Vimentin were significantly upregulated compared to control rats (Fig. 1F). In particular, the levels of TNF-α, IL-1β, PI3K, and p-AKT were elevated in the early CAG stages, indicating active inflammatory signaling. However, at the GC stage, although the expression of these inflammatory factors decreased, proteins associated with tumor progression, including FAK, Itga2, MMP-9, and Vimentin, remained significantly elevated (Fig. 1G–N). This shift in protein expression suggests that inflammation-driven pathways, particularly those involving TNF-α and IL-1β, play a crucial role in initiating gastric carcinogenesis, while tumor-associated signals such as FAK, MMP-9, and Vimentin dominate the later stages, facilitating tumor progression, EMT-associated process, and metastatic potential. These findings provide a comprehensive understanding of the molecular and histopathological evolution of gastric lesions in this rat model, supporting the hypothesis that the inflammatory response in CAG primarily drives tumor initiation, while the later stages of the disease are characterized by tumor-associated signaling pathways that promote malignant transformation and invasiveness.

### Therapeutic effects of RQMJ on inflammation and gastric mucosal lesions in a rat model of CAG

**Fig. 2:**
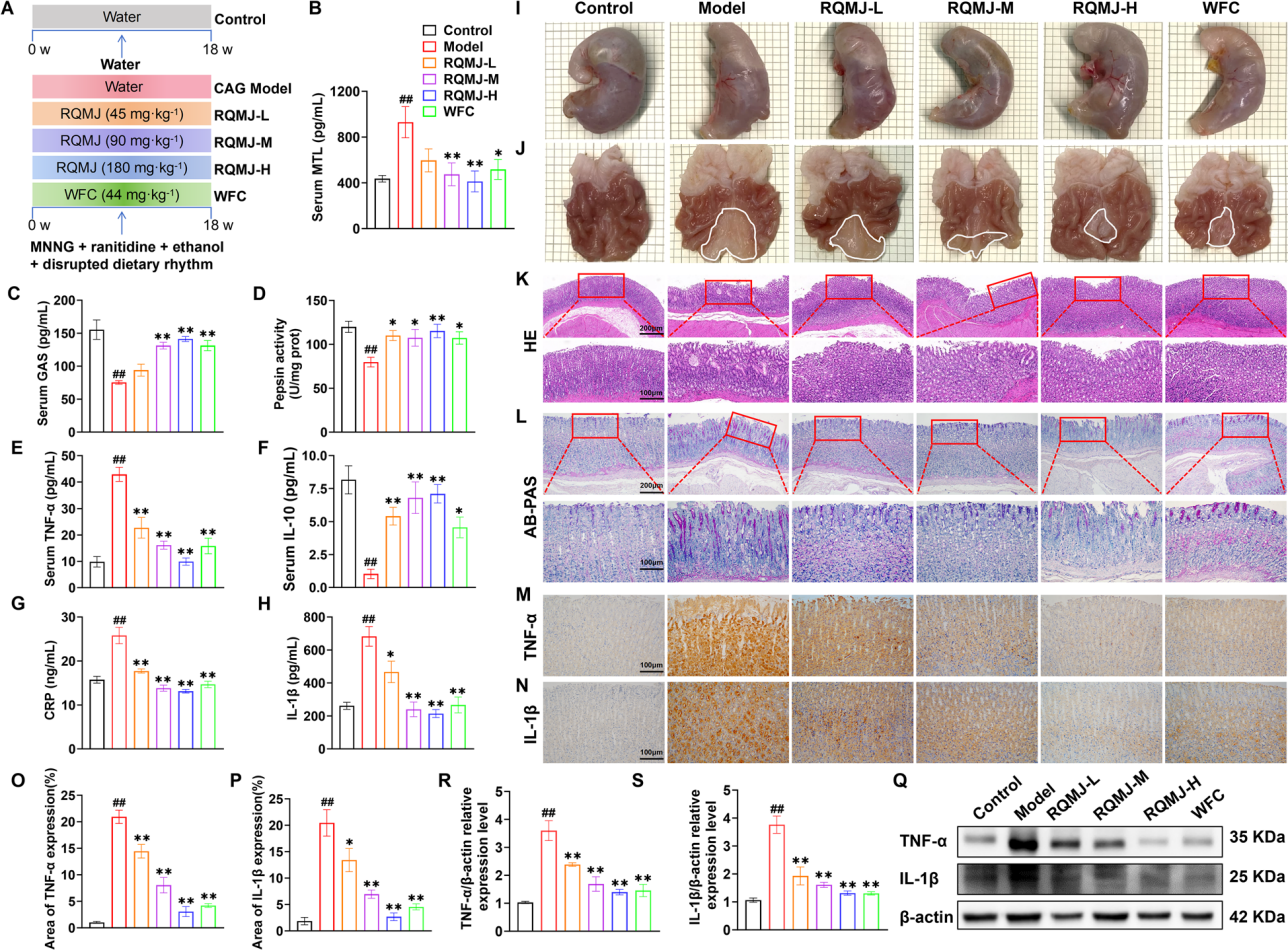
Effects of RQMJ Treatment on Inflammation and Gastric Lesions in CAG Rats. **A** Experimental design and treatment strategy. **B–H** Serum levels of MTL (**B**), GAS (**C**), TNF-α (**E**), IL-10 (**F**), and gastric tissue levels of pepsin activity (**D**), CRP (**G**), IL-1β (**H**) in rats from each group (*n* = 6). **I–J** Gross morphological images of gastric tissues from control, model, WFC, and RQMJ-treated rats at week 18 (*n* = 6). **K–N** Histological examination of gastric tissues via HE staining (**K**), AB-PAS staining (**L**), and immunohistochemical staining for TNF-α (**M**) and IL-1β (**N**) (*n* = 3; scale bars: 200 and 100 μm). **O–P** Quantification of TNF-α and IL-1β immunohistochemical staining areas in the gastric mucosa (*n* = 3). **Q** Western blot analysis of TNF-α and IL-1β protein levels in gastric tissue (*n* = 3). **R–S** Quantification of TNF-α (**R**) and IL-1β (**S**) relative expression levels, with β-actin as the internal control (*n* = 3). Data are presented as mean ± SEM. ^#^*P* < 0.05, ^##^*P* < 0.01, versus the Control group; ^*^P < 0.05, ^**^P < 0.01, versus the Model group respectively

To better evaluate the preventive and therapeutic effects of RQMJ on CAG lesions and to more closely mimic the clinical progression of CAG, a multifactorial combined model (MNNG + ranitidine + ethanol + disrupted dietary rhythm) was established in this study. Pharmacological intervention was initiated concurrently with model induction and continued until week 18. The endpoint was defined as the appearance of typical pathological features of CAG in the model group (Fig. 2A). Body weight was monitored over 18 weeks. As shown in Supplementary Fig.  2 A, rats in the model group exhibited a significant reduction in body weight compared to the control group, while treatment with RQMJ (low, medium, and high doses) and WFC (positive control) alleviated this weight loss, particularly in the medium and high-dose RQMJ groups. ELISA analysis revealed significant changes in gastric function-related markers. MTL levels were significantly decreased, while GAS and pepsin activity levels were significantly increased in the RQMJ-treated groups compared to the model group (Fig. 2B–D). The levels of pro-inflammatory cytokines TNF-α and IL-1β were markedly reduced, whereas the anti-inflammatory cytokine IL-10 was significantly increased in the RQMJ treatment groups, particularly in the high-dose group, compared to the model group (Fig. 2E, F, H). Additionally, CRP levels, which are indicative of systemic inflammation, were significantly reduced in the RQMJ groups (Fig. 2G). The reduction in inflammatory markers suggests that RQMJ treatment helps modulate the inflammatory response in CAG. Histological examination showed that, compared to the control group, model rats exhibited significant gastric mucosal damage with thinning of the mucosa and inflammatory cell infiltration. In the RQMJ-treated groups, particularly at the high dose, the gastric mucosa appeared more intact, with reduced inflammatory infiltration, as shown by HE staining (Fig. 2I–K). AB-PAS staining further confirmed the beneficial effects of RQMJ, as the intestinal metaplasia observed in the model group was significantly reduced in the treatment groups, with less proliferation of goblet cells in the mucosa (Fig. 2L). Immunohistochemical staining for TNF-α and IL-1β showed strong expression in the model group, indicative of persistent inflammation (Fig. 2M, N). The RQMJ-treated rats exhibited significantly reduced TNF-α and IL-1β staining, particularly in the high-dose group, with quantification showing a substantial decrease in the area of positive staining (Fig. 2O, P). To further clarify the regulatory effects of RQMJ on immune cell activation and infiltration, CD68 and CD4 expression in gastric tissues was assessed by immunohistochemical staining. CD68 + macrophages and CD4 + T cells were markedly increased in the model groups, whereas RQMJ treatment reduced their infiltration, suggesting that RQMJ may exert anti-inflammatory effects by inhibiting immune cell activation and inflammatory cytokine release (Supplementary Fig. 2B-E). Western blot analysis corroborated these findings, showing significantly lower levels of TNF-α and IL-1β in the gastric tissues of RQMJ-treated rats compared to the model group (Fig. 2Q–S). These findings demonstrate that RQMJ treatment attenuates the elevated pro-inflammatory cytokines and both systemic and gastric inflammation associated with CAG, thereby helping preserve gastric mucosal integrity. This highlights the crucial role of inflammatory mediators and suggests RQMJ as a potential therapeutic strategy for preventing or treating inflammation-related gastric diseases.

### Therapeutic effects of RQMJ in the inflammation–cancer transition process of CAG

**Fig. 3:**
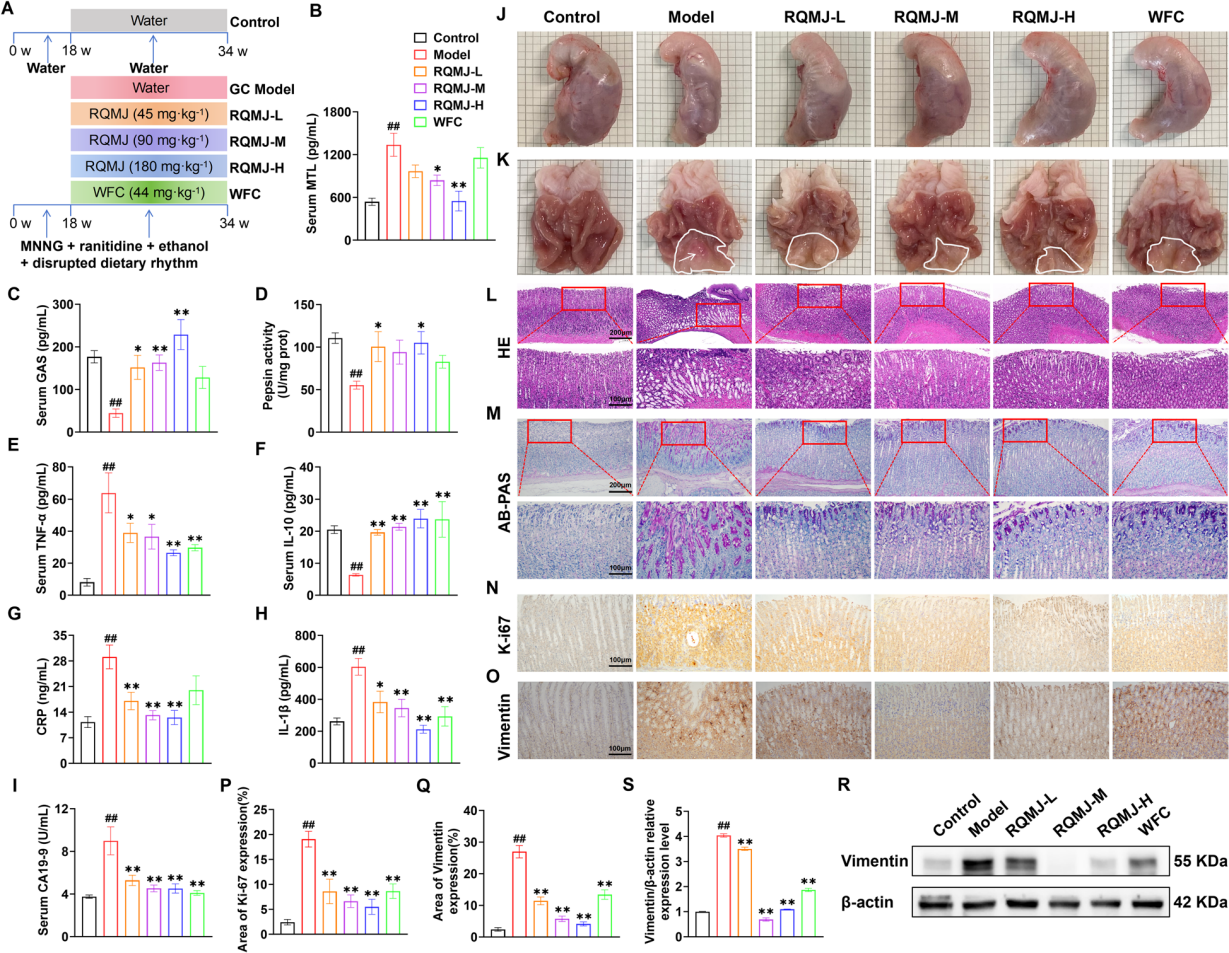
RQMJ intervention in the inflammation–cancer transition of CAG. **A** Experimental design and treatment strategy. **B–H** Serum levels of MTL (**B**), GAS (**C**), TNF-α (**E**), IL-10 (**F**), and gastric tissue levels of pepsin activity (**D**), CRP (**G**), IL-1β (**H**) in rats from each group (*n* = 6). **I** Effect of RQMJ on serum CA19-9 levels in GC rats (*n* = 6). **J** Gross morphological appearance of gastric tissues in each group (*n* = 6). **K** Representative images showing changes in the gastric mucosa across different groups (*n* = 6). **L** Histological examination using HE staining to observe pathological changes in the gastric mucosa (*n* = 3; scale bar: 200 μm), with magnified views of the lesion sites (scale bar: 100 μm). **M** Secretory function of gastric mucosal epithelial cells assessed by AB-PAS staining (*n* = 3, scale bar: 200 μm), with magnified views of the lesion sites (scale bar: 100 μm). **N, P** Immunohistochemical detection of Ki-67 expression in gastric tissues of GC rats following RQMJ intervention (*n* = 3; scale bar: 100 μm). **O, Q** Immunohistochemical detection of Vimentin expression in gastric tissues of GC rats following RQMJ intervention (*n* = 3; scale bar: 100 μm). **R** Western blot analysis of Vimentin protein expression in gastric tissues of GC rats, with β-actin as the internal control (*n* = 3). **S** Densitometric quantification of Western blot bands showing relative expression levels of Vimentin, with β-actin as the internal control (*n* = 3). Data are presented as mean ± SEM. ^#^*P* < 0.05, ^##^*P* < 0.01, versus Control group; ^*^P < 0.05, ^**^P < 0.01, versus Model group

To investigate the effect of RQMJ on the progression of CAG from inflammation to cancer, drug administration was continued from week 18 of modeling to week 34. The endpoint of this phase was defined as the appearance of typical GC pathological features in the model group (Fig. 3A). The therapeutic effects of RQMJ on gastric function, inflammation, and histopathological changes were evaluated in the rat model of GC. Body weight, a key indicator of overall health, was significantly reduced in the model group compared to controls, while RQMJ treatment (particularly at high doses) alleviated weight loss, indicating its potential for improving health (Supplementary Fig.  3 A). ELISA analysis revealed significant changes in gastric function and inflammation markers. Compared with the model group, the RQMJ-treated groups exhibited markedly increased GAS and pepsin activities levels but reduced MTL levels, suggesting that RQMJ can stimulate the secretion of gastric acid and digestive enzymes, and may also have a regulatory effect on gastric motility (Fig. 3B–D). Additionally, pro-inflammatory cytokines such as TNF-α and IL-1β were significantly reduced, whereas the anti-inflammatory cytokine IL-10 was markedly increased in RQMJ-treated groups, particularly at the high dose, indicating that RQMJ effectively modulates the inflammatory response in GC rats (Fig. 3E, F, H). The reduction in CRP levels further supports the anti-inflammatory effects of RQMJ (Fig. 3G). Detection of tumor-associated markers revealed elevated serum CA19-9 levels in GC rats, which were significantly reduced following RQMJ treatment (Fig. 3I). Histopathological analysis revealed severe gastric lesions in the model group, characterized by disorganized and fused glands, marked cellular atypia, nuclear hyperchromasia, and invasive growth. In contrast, RQMJ treatment, particularly at higher doses, prevented the appearance of carcinoma-like features, preserved mucosal integrity, and markedly reduced inflammatory infiltration, as demonstrated by HE staining (Fig. 3J–L). AB-PAS staining confirmed that pronounced intestinal metaplasia, increased mucin secretion, elevated goblet cell numbers, and disrupted mucosal architecture observed in the model group were markedly reduced following RQMJ treatment, with goblet cell numbers in the mucosa significantly decreased (Fig. 3M). Immunohistochemical staining for Ki-67 and vimentin revealed strong expression in GC rats, indicating persistent tumor proliferation and epithelial–mesenchymal characteristics (Fig. 3N–O). In contrast, RQMJ-treated rats, particularly those in the high-dose group, showed markedly reduced staining intensity (Fig. 3P–Q). Furthermore, CD68 and CD4 staining demonstrated CD68 + macrophages and CD4 + T cells were markedly increased in the model groups, whereas RQMJ treatment reduced their infiltration (Supplementary Fig. 3B-E). Western blot analysis corroborated these findings, with significantly lower levels of Vimentin in the gastric tissues of RQMJ-treated rats compared to the GC rats (Fig. 3R–S). Collectively, these findings indicate that RQMJ delays the progression of inflammation–cancer transition in rats, likely by reducing pro-inflammatory cytokines, which in turn suppresses downstream tumor-associated molecular markers and improves gastric function.

### RQMJ inhibits MNNG-induced malignant transformation of gastric mucosal epithelial cells

**Fig. 4:**
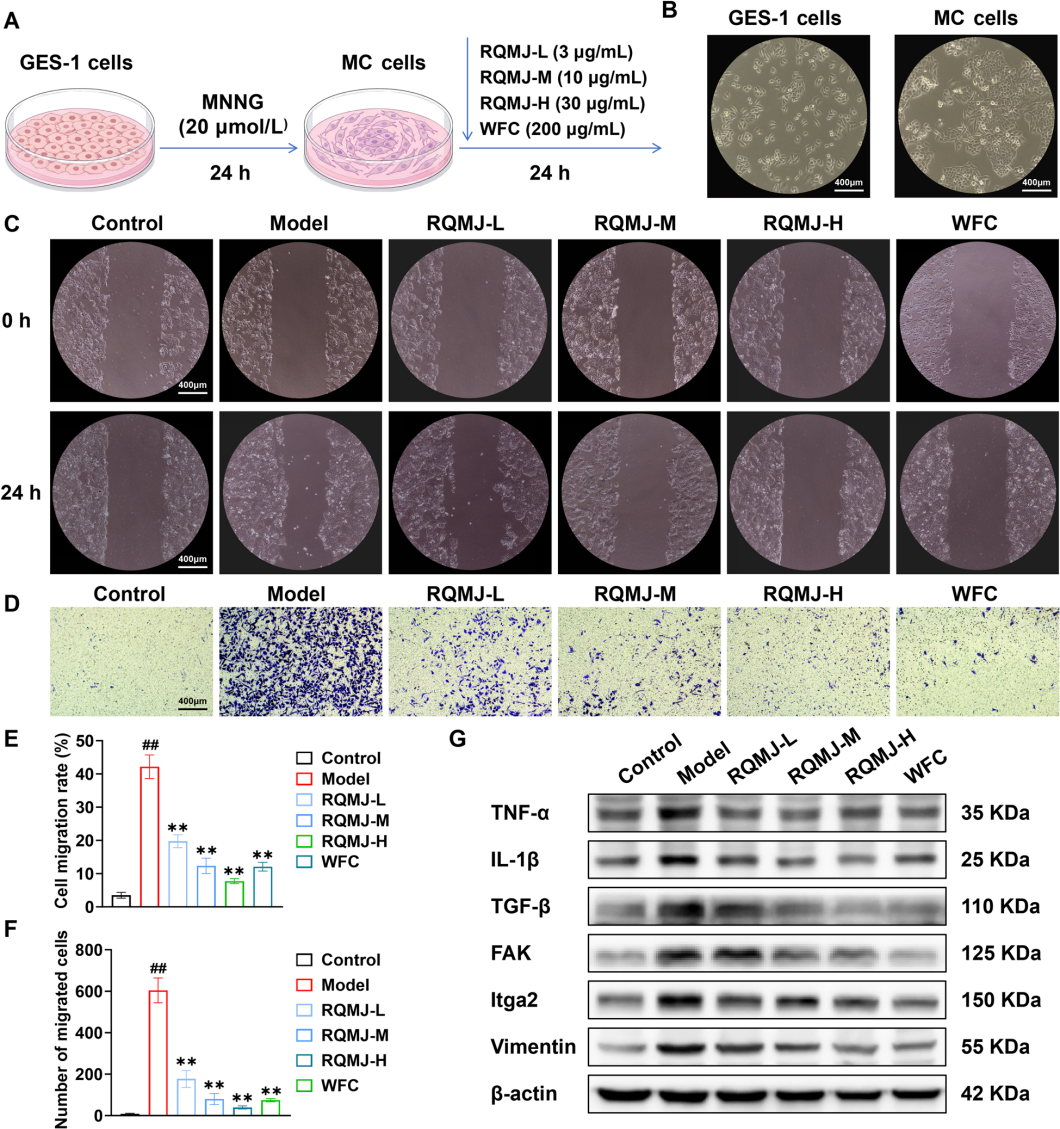
RQMJ inhibits MNNG-induced malignant transformation of gastric mucosal epithelial cells. **A** Experimental design and treatment strategy. **B** Morphological changes of GES-1 cells after 24 h of MNNG induction (n = 3; scale bar: 400 μm). **C** Wound-healing assay showing the effect of RQMJ on MC cell migration (n = 3; scale bar: 400 μm). **D** Transwell assay showing the effect of RQMJ on MC cell migration (n = 3; scale bar: 400 μm). **E–F** Quantification of wound closure and migrated cells after RQMJ treatment (n = 3). **G** Western blot analysis of TNF-α, IL-1β, TGF-β, FAK, Itga2, and Vimentin in MC cells, with β-actin as the loading control (n = 3). Data are presented as mean ± SEM. ^##^*P* < 0.01, versus Control group; ^**^P < 0.01, versus Model group

To evaluate the effects of RQMJ on the malignant transformation of gastric mucosal epithelial cells, MC cells were established by MNNG-induced transformation of normal GES-1 cells (Fig. 4A). Following induction, cells exhibited marked morphological changes, transitioning from epithelial-like morphology to polygonal or spindle-shaped cells with pseudopodia-like extensions. The cells became elongated and disorganized, with a “spiky” peripheral appearance, increased cytoplasmic vacuolization, enhanced intercellular contacts, and island-like clustered growth surrounded by spindle-shaped cells (Fig. 4B). Scratch wound-healing assays demonstrated that RQMJ significantly inhibited MC cell migration and proliferation, with the high-dose group (RQMJ-H) showing the slowest wound closure rate (Fig. 4C, E). Transwell migration assays further confirmed that RQMJ markedly reduced MC cell migratory capacity in a dose-dependent manner, with the strongest inhibitory effect observed in the high-dose group (Fig. 4D, F). Western blot analysis showed that RQMJ significantly downregulated the expression of inflammation- and EMT-related proteins, including TNF-α, IL-1β, TGF-β, FAK, Itga2, and Vimentin, compared with the model group (Fig. 4G and Supplementary Fig. 4A-F). Collectively, these findings indicate that RQMJ effectively suppresses the malignant phenotype of MC cells, including significant inhibition of cell proliferation and migration, and may partially reverse inflammation-associated cellular transformation.

### Transcriptome analysis reveals key pathways modulated by RQMJ in CAG Rats

**Fig. 5:**
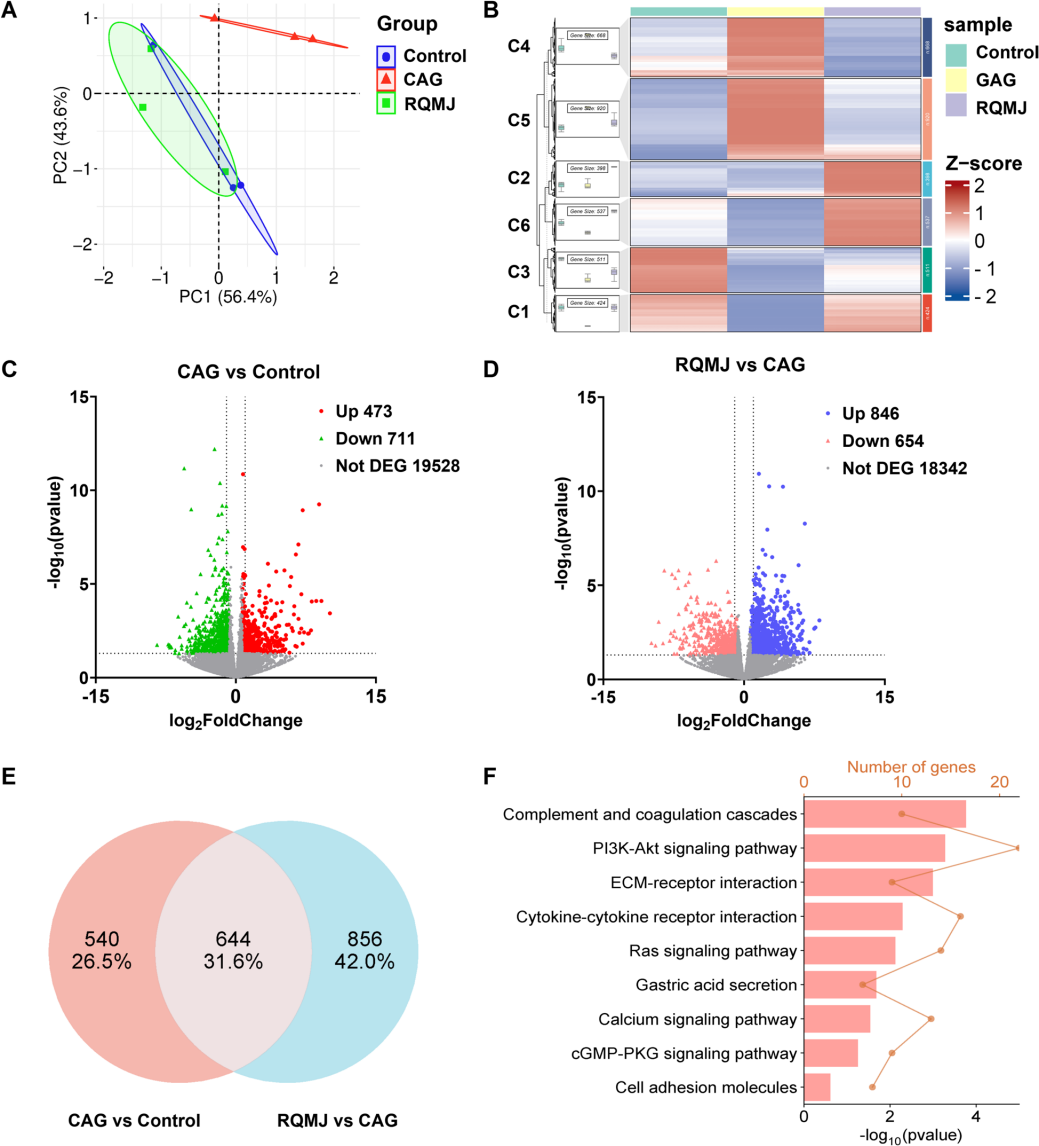
RNA-seq analysis of gene expression in the rat Model of CAG and the effects of RQMJ treatment. **A** PCA plot showing the clustering of Control, CAG, and RQMJ-treated groups based on gene expression profiles. **B** Heatmap of the DEGs between Control, CAG, and RQMJ-treated groups, with Z-scores representing relative expression levels. **C** Volcano plot comparing gene expression between CAG and control groups, highlighting upregulated (red) and downregulated (green) genes. **D** Volcano plot comparing gene expression between RQMJ and CAG groups, highlighting upregulated (blue) and downregulated (pink) genes. **E** Venn diagram showing the overlap of DEGs between CAG vs. control and RQMJ vs. CAG comparisons. **F** KEGG pathway analysis of 644 DEGs

To explore the molecular mechanisms underlying the effects of RQMJ in the CAG rat model, RNA-seq was performed to analyze gene expression profiles. PCA revealed distinct clustering of the control, CAG, and RQMJ-treated groups, whereas the Control and RQMJ groups showed minimal separation, indicating that RQMJ treatment alters the gene expression profile in CAG rats, bringing it closer to the control group (Fig. 5A). A heatmap of the DEGs between the three groups showed distinct gene expression patterns in the control, CAG, and RQMJ-treated groups, with significant changes observed in the RQMJ-treated group compared to the model group (Fig. 5B). Volcano plots further illustrated the number of upregulated and downregulated genes in the comparisons of CAG vs. control and RQMJ vs. CAG. In the CAG vs. control comparison, 473 genes were upregulated, and 711 genes were downregulated (Fig. 5C). In the RQMJ vs. CAG comparison, 846 genes were upregulated, and 654 genes were downregulated (Fig. 5D). A Venn diagram showed that 644 DEGs were shared between the CAG vs. control and RQMJ vs. CAG comparisons, indicating common pathways and molecular mechanisms involved in both CAG progression and RQMJ treatment (Fig. 5E). KEGG pathway enrichment analysis identified several significantly enriched pathways in the RQMJ vs. CAG comparison, including PI3K–AKT signaling pathway, extracellular matrix (ECM)-receptor interaction, cGMP–PKG signaling pathway, and gastric acid secretion (Fig. 5F). These pathways are known to play crucial roles in inflammation, cell migration, and tumor progression, supporting the notion that RQMJ modulates key signaling networks involved in the inflammation-cancer transition.

### RQMJ modulates CAG inflammation–cancer transition via the cGMP–PKG/PI3K–AKT signaling pathway

**Fig. 6:**
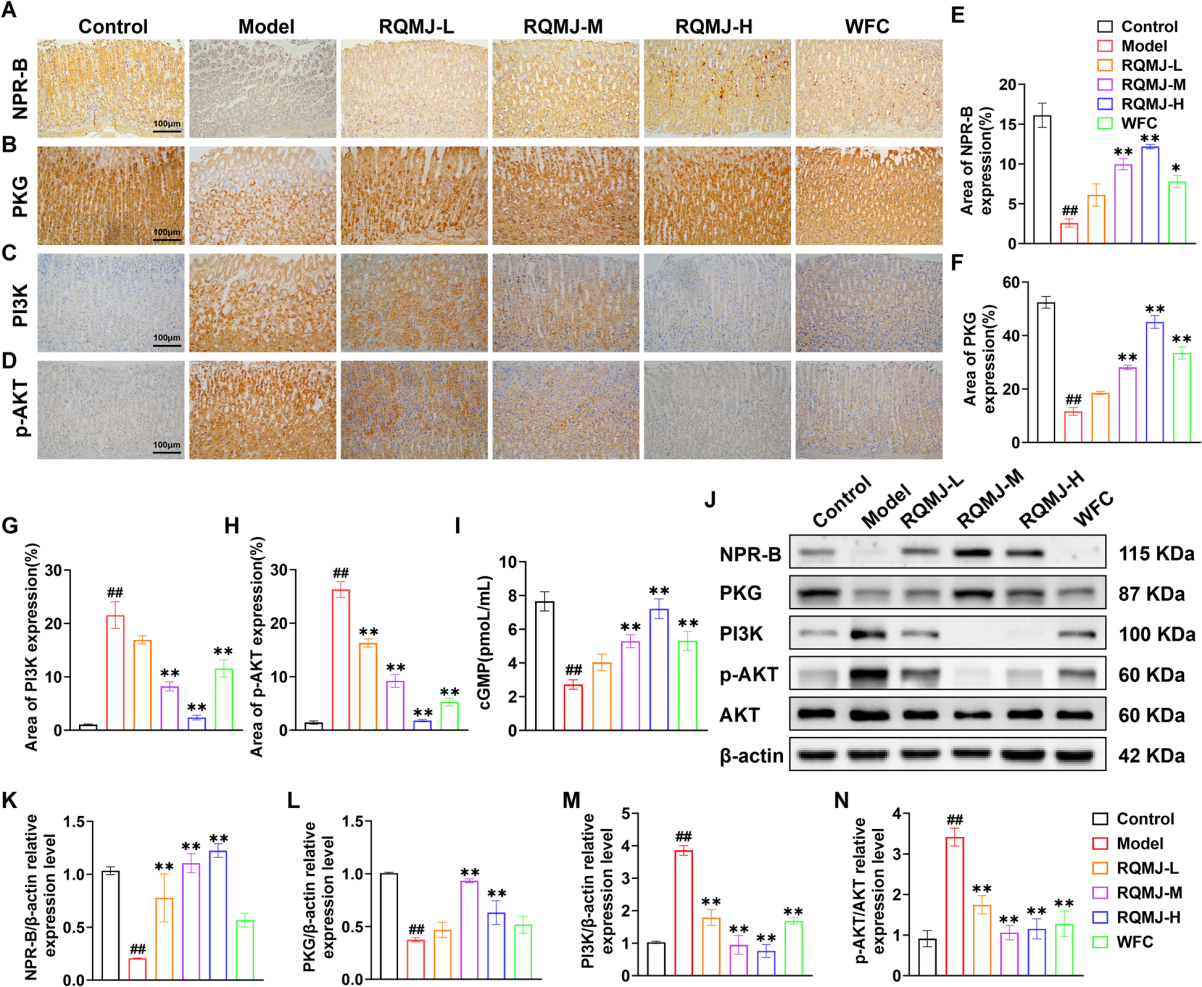
Effects of RQMJ on the Expression of NPR-B, PKG, PI3K, and p-AKT in the Gastric Tissues of CAG Rats. **A–D** Immunohistochemical staining for NPR-B (**A**), PKG (**B**), PI3K (**C**), and p-AKT (**D**) in gastric tissues from control, model, and RQMJ-treated groups (*n* = 3; scale bar: 100 μm). **E–H** Quantification of the area of expression for NPR-B, PKG, PI3K, and p-AKT in gastric tissues (*n* = 3). **I** Measurement of serum cGMP levels in CAG rats by ELISA (*n* = 6). **J** Western blot analysis of NPR-B, PKG, PI3K, p-AKT, and AKT protein levels in gastric tissues (*n* = 3). **K–N** Quantification of the relative expression levels of NPR-B, PKG, PI3K, p-AKT, and AKT from Western blot data. Data are presented as mean ± SEM. ^##^*P* < 0.01, versus the Control group; ^*^P < 0.05, ^**^P < 0.01, versus the Model group

**Fig. 7:**
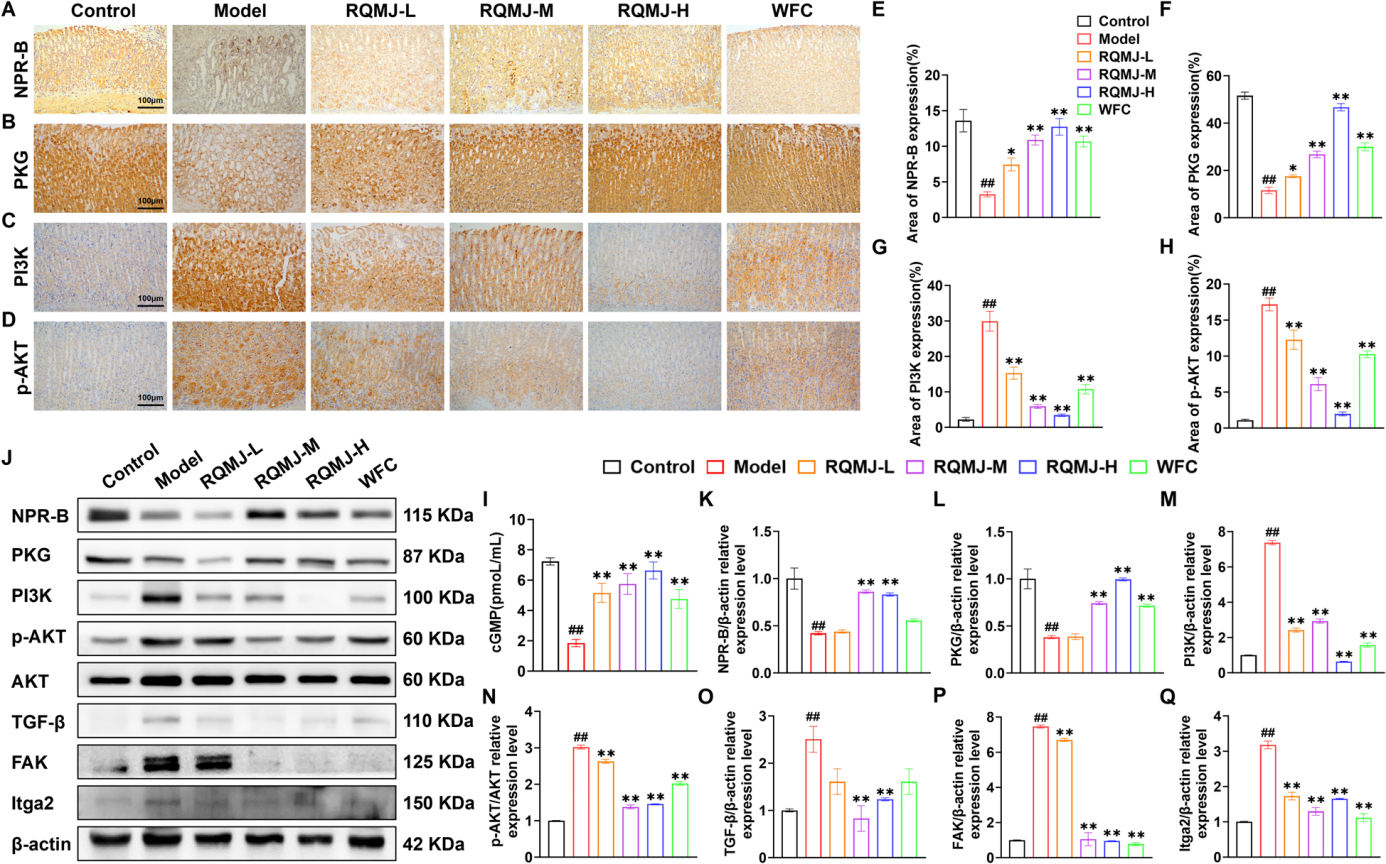
Effects of RQMJ on NPR-B, PKG, PI3K, p-AKT, and Related Signaling Pathways in GC Rats. **A–D** Immunohistochemical staining for NPR-B (**A**), PKG (**B**), PI3K (**C**), and p-AKT (**D**) in gastric tissues from control, model, and RQMJ-treated groups (*n* = 3; scale bar: 100 μm). **E–H** Quantification of the area of expression for NPR-B, PKG, PI3K, and p-AKT in gastric tissues, showing significant differences between the model and RQMJ-treated groups (*n* = 3). **I** Measurement of serum cGMP levels in GC rats by ELISA (*n* = 6). **J** Western blot analysis of NPR-B, PKG, PI3K, p-AKT, AKT, TGF-β, FAK, and Itga2 protein levels in gastric tissues (*n* = 3). **K–Q** Quantification of the relative expression levels of NPR-B, PKG, PI3K, p-AKT, TGF-β, FAK, and Itga2 from Western blot data. Data are presented as mean ± SEM. ^##^*P* < 0.01, versus the Control group; ^*^P < 0.05, ^**^P < 0.01, versus the Model group

The effects of RQMJ on inflammation and key signaling pathways in gastric tissues of CAG and GC rats were evaluated using immunohistochemical, ELISA, and Western blot. Immunohistochemical staining revealed CAG and GC rats exhibited significantly decreased expression of NPR-B and PKG, whereas PI3K and p-AKT were markedly upregulated, compared to controls, indicating active signaling in the gastric tissue (Fig. 6, 7A–D). Treatment with RQMJ or WFC, NPR-B, and PKG expression were significantly increased, while PI3K and p-AKT expression were significantly reduced (Fig. 6, 7E–H). ELISA results showed that, compared with the control group, cGMP levels were significantly reduced in the model group, whereas RQMJ treatment markedly increased cGMP levels (Fig. 6, 7I). Western blot analysis confirmed these findings, showing significantly decreased expression of NPR-B and PKG, whereas PI3K and p-AKT were markedly upregulated in the model group compared to controls (Fig. 6, 7J). RQMJ treatment significantly reversed the expression of these proteins (Fig. 6, 7K–N). These results indicate that RQMJ treatment activates the cGMP–PKG signaling axis and concurrently suppresses the PI3K–Akt pathway, ultimately regulating inflammation- and proliferation-related processes. In addition to inflammatory signaling proteins, Western blot also revealed significant changes in proteins associated with fibrosis and cell migration. The expression of TGF-β, FAK, and Itga2 was significantly upregulated in the GC model group, suggesting the activation of pathways involved in fibrosis, EMT-related changes, and GC progression (Fig. 7J). Treatment with RQMJ significantly reduced the expression of these proteins (Fig. 7O–Q), indicating that RQMJ may inhibit fibrosis and EMT-related changes, thus preventing the malignant transformation of gastric tissue. Taken together, these findings suggest that RQMJ may modulate the tumor microenvironment and intervene in the inflammation–cancer transition by activating the cGMP–PKG signaling pathway, inhibiting its downstream PI3K–AKT pathway, and regulating ECM–receptor interactions, suggesting its potential as a therapeutic strategy for preventing gastric inflammation–cancer transition.

### RQMJ inhibits malignant transformation of gastric mucosal epithelial cells via the cGMP–PKG/PI3K–AKT signaling pathway

**Fig. 8:**
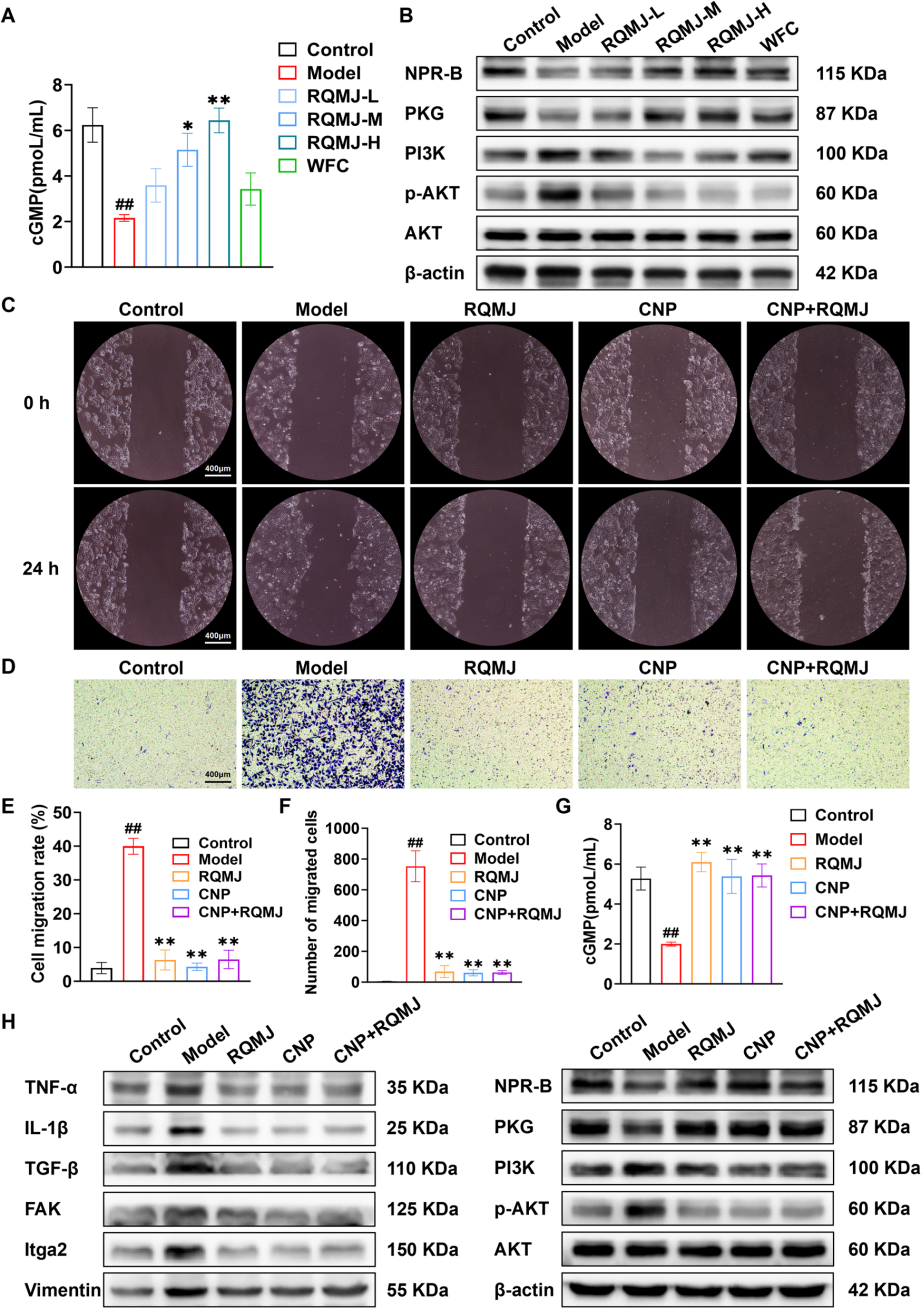
RQMJ inhibits malignant transformation of gastric mucosal epithelial cells via the cGMP–PKG/PI3K–AKT signaling pathway. **A** Cellular cGMP levels in MC cells measured by ELISA (n = 6). **B** Western blot analysis of NPR-B, PKG, PI3K, p-AKT, and total AKT in MC cells (n = 3). **C** Wound-healing assay showing the effects of RQMJ and/or CNP on MC cell migration (n = 3; scale bar: 400 μm). **D** Transwell assay showing the effects of RQMJ and/or CNP on MC cell migration (n = 3; scale bar: 400 μm). **E–F** Quantification of wound closure and migrated cells (n = 3). **G** Cellular cGMP levels after RQMJ and/or CNP treatment measured by ELISA (n = 6). **H** Western blot analysis of TNF-α, IL-1β, TGF-β, FAK, Itga2, Vimentin, NPR-B, PKG, PI3K, p-AKT, and total AKT in MC cells (n = 3). Data are presented as mean ± SEM. ^##^*P* < 0.01, versus the Control group; ^*^P < 0.05, ^**^P < 0.01, versus the Model group

We investigated whether the cGMP–PKG/PI3K–AKT signaling pathway mediates the protective effects of RQMJ on gastric epithelial malignant transformation. ELISA results showed that, compared with the control group, cGMP levels were significantly reduced in the model group, whereas RQMJ treatment markedly restored cGMP levels (Fig. 8A). Western blot further confirmed these findings, showing decreased expression of NPR-B and PKG, accompanied by increased expression of PI3K and p-AKT in the model group compared with controls. These changes were significantly reversed by RQMJ treatment (Fig. 8B and Supplementary Fig. 5A-D), suggesting that RQMJ inhibits MC cell malignant transformation potentially via activation of the cGMP–PKG signaling pathway. To further validate the role of the cGMP–PKG pathway in mediating the effects of RQMJ, CNP, a selective NPR-B agonist that promotes cGMP production and activates PKG-dependent signaling, was employed. Wound-healing and Transwell assays demonstrated that both RQMJ and CNP significantly inhibited MC cell migration and proliferation (Fig. 8C–F). Notably, under CNP intervention, the inhibitory effects of RQMJ on MC cell migration and proliferation were not further enhanced. ELISA and Western blot showed that RQMJ increased intracellular cGMP levels, reversed model-induced changes in NPR-B, PKG, PI3K, and p-AKT, and reduced the expression of TNF-α, IL-1β, TGF-β, FAK, Itga2, and Vimentin. CNP produced a similar regulatory pattern. Importantly, when the pathway was already activated by CNP, co-treatment with RQMJ did not further increase NPR-B/cGMP/PKG levels or further suppress PI3K/p-AKT (Fig. 8G, H and Supplementary Fig. 5I–N). This non-additive pattern supports the interpretation that RQMJ acts, through the cGMP–PKG signaling pathway and its downstream modulation of PI3K–AKT signaling pathway.

## Discussion

GC remains a highly prevalent and deadly gastrointestinal malignancy, marked by its invasiveness and metastatic potential. Despite advances in early detection, helicobacter pylori eradication, and therapeutic strategies like surgery, chemotherapy, targeted therapy, and immunotherapy, most GC cases are diagnosed at advanced stages, limiting therapeutic efficacy and survival outcomes [20]. The pathogenesis of GC follows a multistage process, commonly described by the Correa cascade, in which CAG plays a pivotal role as the key inflammatory stage leading to malignant transformation [21]. CAG is characterized by glandular loss, impaired mucosal barrier function, persistent inflammation, and intestinal metaplasia, which contribute to the initiation and progression of GC. The inflammatory microenvironment is crucial in this transformation, driving oncogene activation, tumor suppressor gene inactivation, and promoting uncontrolled cell proliferation [22].

Effective intervention at the CAG stage is essential to delay, block, or reverse the inflammation-driven malignant transformation, thus reducing the risk of GC. However, research on CAG and its progression to GC has been limited by the lack of standardized modeling methods. Traditional single-factor models, such as MNNG-induced CAG, fail to fully replicate the complex, multifactorial pathogenesis seen in humans. To better mimic the clinical progression of CAG, our study utilized a multifactorial combined model (MNNG + ranitidine + ethanol + disrupted dietary rhythm), which faithfully reproduced the key aspects of gastric mucosal injury, acid suppression, mucosal barrier dysfunction, and dietary rhythm disruption [23]. Dynamic sampling every two weeks and histopathological evaluation demonstrated that this model recapitulated the natural progression from CAG to GC, with clear pathological stage specificity and controllability. By week 18, rats exhibited typical features of CAG, including thinning gastric mucosa, disordered glandular structures, and inflammatory infiltration. The PI3K–AKT and FAK pathways were activated, and EMT was induced, collectively enhancing cellular migratory capacity and exacerbating the inflammatory response. By week 34, dysplasia and tumor masses were visible, with the model group progressing to GC. These findings demonstrate that our model accurately reflects the chronic inflammation–cancer transition process and provides a valuable platform for testing therapeutic interventions.

RQMJ, a classical Tibetan medicine, has long been used for treating chronic gastritis due to its therapeutic properties, including anti-inflammatory effects, gastric mucosal nourishment, and hepatoprotection. In the present study, a comprehensive chemical characterization of RQMJ was performed using UPLC-MS/MS, resulting in the identification of 2780 chemical constituents, which provides an important foundation for elucidating its pharmacodynamic material basis and guiding future investigations of its bioactive components. Given the multi-component, multi-target, and multi-pathway characteristics of Tibetan medicinal formulations, the therapeutic effects of RQMJ are more likely attributable to the synergistic actions of multiple bioactive constituents rather than a single compound. Therefore, the present study adopted a whole-formula intervention strategy to systematically evaluate the effects of RQMJ on CAG and its inflammation–cancer transition process. The results indicated that RQMJ significantly improved the pathological architecture of the gastric mucosa, reduced inflammatory cell infiltration, and downregulated pro-inflammatory mediators such as TNF-α, IL-1β, and CRP. Furthermore, RQMJ restored gastrointestinal function by modulating gastric factors such as GAS, MTL, and pepsin activity. These findings suggest that RQMJ exerts both anti-inflammatory and mucosal restorative effects. Moreover, RQMJ treatment significantly reduced tumor-associated markers such as Ki-67, vimentin, and CA19-9, suggesting that RQMJ interferes with the inflammation–cancer transition of CAG and delays the progression to GC by regulating the balance between cell proliferation and apoptosis. Consistently, in vitro experiments further confirmed that RQMJ significantly inhibited the proliferation and migration of MC cells, thereby suppressing the malignant phenotype of gastric epithelial cells.

The mechanisms underlying the inflammation–cancer transition are complex, involving oxidative stress, DNA damage, defective repair, and the upregulation of pro-inflammatory cytokines like TNF-α and IL-1β. Key signaling pathways such as NF-κB, PI3K–AKT, and MEK–ERK are activated, facilitating the progression of inflammation to cancer and promoting EMT-associated process [24–27]. To investigate the molecular mechanisms by which RQMJ regulates this transition, we conducted RNA-seq to examine transcriptomic changes in gastric tissues [28]. Our findings revealed that RQMJ’s inhibitory effects are closely linked to the activation of the cGMP–PKG pathway, modulation of the PI3K–AKT pathway, and regulation of ECM–receptor interactions. NPR-B, a guanylyl cyclase receptor expressed primarily in the gastric mucosa, catalyzes the conversion of GTP to cGMP, which activates downstream PKG signaling [29, 30]. PKG signaling has been shown to suppress PI3K–AKT activity, reducing cell proliferation and promoting mucosal repair [31, 32]. In GC, the PI3K–AKT pathway activates MMP-2/9, promoting ECM remodeling, cell migration, and immune evasion, thus facilitating tumor progression [33, 34]. Our in vivo and in vitro experimental data suggest that RQMJ may inhibit inflammatory factors by activating the cGMP–PKG pathway and suppressing the PI3K–AKT signaling, thereby modulating ECM–receptor interactions and preventing inflammation–cancer transition.

Although the present study provides important evidence supporting the protective effects of RQMJ against CAG and its inflammation–cancer transition, several limitations should be acknowledged. The bioactive constituents directly responsible for NPR-B activation remain unclear. Previous studies suggest that representative compounds in RQMJ, particularly saffron-derived crocins, possess anti-inflammatory, anti-tumor, and gastroprotective activities and regulate the PI3K–AKT signaling pathway [35]. Future studies will combine activity-guided fractionation, targeted validation of representative compounds, and compound–pathway correlation analysis to clarify the pharmacodynamic basis of RQMJ, while further examining canonical EMT markers, including E-cadherin, N-cadherin, Snail, and ZEB1. In addition, although RQMJ showed significant therapeutic efficacy in animal and cellular models, comprehensive toxicological evaluation was beyond the scope of this study. Therefore, its long-term safety and clinical efficacy require further validation through toxicological studies and well-designed clinical trials.

## Conclusion

In summary, this study confirms that RQMJ exerts significant therapeutic effects against CAG and may delay its progression to GC. Through its anti-inflammatory actions, RQMJ appears to suppress inflammatory factors by activating the cGMP–PKG pathway and inhibiting the PI3K–AKT signaling cascade, thereby regulating ECM–receptor interactions and the EMT process, and thus indirectly preventing malignant transformation to GC. These findings underscore its potential as a therapeutic strategy for inflammation-driven gastric diseases.

## Supplementary Information

Supplementary Material 1

Supplementary Material 2

Supplementary Material 3

## Data Availability

All data and materials during the current study are available from the corresponding author upon reasonable request.

## Abbreviations

GC: Gastric cancer
CAG: Chronic atrophic gastritis
RQMJ: Renqing Mangjue
ELISA: Enzyme-linked immunosorbent assay
HE: Hematoxylin and eosin
AB-PAS: Alcian blue–periodic acid–Schiff
PI3K: Phosphoinositide 3-kinase
AKT: Protein kinase B
FAK: Focal adhesion kinase
EMT: Epithelial–mesenchymal transition
TNF-α: Tumor necrosis factor-alpha
IL-1β: Interleukin-1 beta
CRP: C-reactive protein
IL-10: Interleukin-10
CA19-9: Carbohydrate antigen 19–9
NPR-B: Natriuretic peptide receptor-B
cGMP: Cyclic guanosine monophosphate
PKG: cGMP-dependent protein kinase
TGF-β: Transforming growth factor-beta
Itga2: Integrin alpha-2
MEK: Mitogen-activated protein kinase kinase
ERK: Extracellular signal-regulated kinase
GSK-3β: Glycogen synthase kinase-3 beta
MMP-9: Matrix metalloproteinase-9
NF-κB: Nuclear factor kappa-B
ECM: Extracellular matrix
WFC: Weifuchun
MNNG: N-methyl-N′-nitro-N-nitrosoguanidine
GAS: Gastrin
MTL: Motilin
RIPA: Radioimmunoprecipitation assay
β-actin: Beta-actin
BSA: Bovine serum albumin

## References

[1] Jia R, Guo X, Liu H, et al. Analysis of staged features of gastritis-cancer transformation and identification of potential biomarkers in gastric cancer. J Inflamm Res. 2022;15:6857–68. 10.2147/jir.s390448. 36597437 10.2147/JIR.S390448 PMC9805741

[2] Smyth EC, Nilsson M, Grabsch HI, et al. Gastric cancer. Lancet. 2020;396(10251):635–48. 10.1016/s0140-6736(20)31288-5. 32861308 10.1016/S0140-6736(20)31288-5

[3] Guo H, Tang H, Zhao Y, et al. Molecular typing of gastric cancer based on invasion-related genes and prognosis-related features. Front Oncol. 2022;12:848163. 10.3389/fonc.2022.848163. 35719914 10.3389/fonc.2022.848163 PMC9203697

[4] Correa P. Human gastric carcinogenesis: a multistep and multifactorial process–First American Cancer Society Award Lecture on Cancer Epidemiology and Prevention. Cancer Res. 1992;52(24):6735–40. 1458460

[5] Li J, Pan J, Xiao D, et al. Chronic atrophic gastritis and risk of incident upper gastrointestinal cancers: a systematic review and meta-analysis. J Transl Med. 2024;22(1):429. 10.1186/s12967-023-04736-w. 38711123 10.1186/s12967-023-04736-w PMC11075312

[6] Shah SC, Piazuelo MB, Kuipers EJ, et al. AGA Clinical practice update on the diagnosis and management of atrophic gastritis: expert review. Gastroenterology. 2021;161(4):1325-1332.e7. 10.1053/j.gastro.2021.06.078. 34454714 10.1053/j.gastro.2021.06.078 PMC8740554

[7] Dhondup L, Husted C. Tibetan medicine and regeneration. Ann Ny Acad Sci. 2009;1172:115–22. 10.1111/j.1749-6632.2009.04500.x. 19735245 10.1111/j.1749-6632.2009.04500.x

[8] Husted C, Dhondup L. Tibetan medical interpretation of myelin lipids and multiple sclerosis. Ann Ny Acad Sci. 2009;1172:278–96. 10.1196/annals.1393.022. 19743559 10.1196/annals.1393.022

[9] Dhondrup R, Tidwell T, Zhang X, et al. Tibetan medicine Liuwei Muxiang pills (LWMX pills) effectively protects mice from chronic non-atrophic gastritis. Phytomedicine. 2023;115:154826. 10.1016/j.phymed.2023.154826. 37167846 10.1016/j.phymed.2023.154826

[10] Xu C, Rezeng C, Li J, et al. 1H NMR-based metabolomics study of the toxicological effects in rats induced by “Renqing Mangjue” Pill, a Traditional Tibetan Medicine. Front Pharmacol. 2017;8:602. 10.3389/fphar.2017.00602. 28928660 10.3389/fphar.2017.00602 PMC5591455

[11] Liu XN, Shi XY, Jia SY, et al. Rapid multi-elemental analysis on four precious Tibetan medicines based on LIBS technique. China J Chin Mater Med. 2015;40(11):2239–43. 26552188

[12] Greten FR, Grivennikov SI. Inflammation and Cancer: Triggers, Mechanisms, and Consequences. Immunity. 2019;51(1):27–41. 10.1016/j.immuni.2019.06.025. 31315034 10.1016/j.immuni.2019.06.025 PMC6831096

[13] Zhao H, Wu L, Yan G, et al. Inflammation and tumor progression: signaling pathways and targeted intervention. Signal Transduct Tar. 2021;6(1):263. 10.1038/s41392-021-00658-5. 10.1038/s41392-021-00658-5 PMC8273155 34248142

[14] Fernandes Q, Inchakalody VP, Bedhiafi T, et al. Chronic inflammation and cancer; the two sides of a coin. Life Sci. 2023. 10.1016/j.lfs.2023.122390. 38160787 10.1016/j.lfs.2023.122390

[15] Chu F, Li Y, Meng X, et al. Gut Microbial Dysbiosis and changes in fecal metabolic phenotype in precancerous lesions of gastric cancer induced with N-Methyl-N’-Nitro-N-Nitrosoguanidine, Sodium Salicylate, Ranitidine, and Irregular diet. Front Physiol. 2021;12:733979. 10.3389/fphys.2021.733979. 34803728 10.3389/fphys.2021.733979 PMC8599278

[16] Tang D, Chen M, Huang X, et al. SRplot: A free online platform for data visualization and graphing. PLoS ONE. 2023;18(11):e0294236. 10.1371/journal.pone.0294236. 37943830 10.1371/journal.pone.0294236 PMC10635526

[17] Dennis G, Sherman BT, Hosack DA, et al. DAVID: Database for Annotation, Visualization, and Integrated Discovery. Genome Biol. 2003;4(5):P3.12734009

[18] Cai J, Wang M, Zhu M, et al. N-methyl-N-nitro-N’-nitrosoguanidine induces the expression of CCR2 in human gastric epithelial cells promoting CCL2-mediated migration. Mol Med Rep. 2016;13(2):1083–90. 10.3892/mmr.2015.4650. 26648448 10.3892/mmr.2015.4650 PMC4732851

[19] Zeng R, Gou H, Lau HCH, et al. Stomach microbiota in gastric cancer development and clinical implications. Gut. 2024;73(12):2062–73. 10.1136/gutjnl-2024-332815. 38886045 10.1136/gutjnl-2024-332815 PMC11672014

[20] Sundar R, Nakayama I, Markar SR, et al. Gastric cancer. Lancet. 2025;405(10494):2087–102. 10.1016/s0140-6736(25)00052-2. 40319897 10.1016/S0140-6736(25)00052-2

[21] Piazuelo MB, Bravo LE, Mera RM, et al. The Colombian Chemoprevention Trial: 20-Year Follow-Up of a cohort of patients with gastric precancerous lesions. Gastroenterology. 2021;160(4):1106-1117.e3. 10.1053/j.gastro.2020.11.017. 33220252 10.1053/j.gastro.2020.11.017 PMC7956231

[22] Kuang W, Xu J, Xu F, et al. Current study of pathogenetic mechanisms and therapeutics of chronic atrophic gastritis: a comprehensive review. Front Cell Dev Biol. 2024;12:1513426. 10.3389/fcell.2024.1513426. 39720008 10.3389/fcell.2024.1513426 PMC11666564

[23] Zu GX, Sun QQ, Chen J, et al. Urine metabolomics of rats with chronic atrophic gastritis. PLoS ONE. 2020;15(11):e0236203. 10.1371/journal.pone.0236203. 33175875 10.1371/journal.pone.0236203 PMC7657567

[24] Akkız H, Şimşek H, Balcı D, et al. Inflammation and cancer: molecular mechanisms and clinical consequences. Front Oncol. 2025;15:1564572. 10.3389/fonc.2025.1564572. 40165901 10.3389/fonc.2025.1564572 PMC11955699

[25] Jaroenlapnopparat A, Bhatia K, Coban S. Inflammation and gastric cancer. Diseases. 2022;10(3):35. 10.3390/diseases10030035. 35892729 10.3390/diseases10030035 PMC9326573

[26] Nishida A, Andoh A. The Role of inflammation in cancer: mechanisms of tumor initiation, progression, and metastasis. Cells. 2025;14(7):488. 10.3390/cells14070488. 40214442 10.3390/cells14070488 PMC11987742

[27] Oh A, Pardo M, Rodriguez A, et al. NF-κB signaling in neoplastic transition from epithelial to mesenchymal phenotype. Cell Commun Signal. 2023;21(1):291. 10.1186/s12964-023-01207-z. 37853467 10.1186/s12964-023-01207-z PMC10585759

[28] Ren F, Wei J, Chen Q, et al. Artificial intelligence-driven multi-omics approaches in Alzheimer’s disease: Progress, challenges, and future directions. Acta Pharm Sin B. 2025;15(9):4327–85. 10.1016/j.apsb.2025.07.030. 41049729 10.1016/j.apsb.2025.07.030 PMC12491694

[29] Gower WR, Salhab KF, Foulis WL, et al. Regulation of atrial natriuretic peptide gene expression in gastric antrum by fasting. Am J Physiol-Reg I. 2000;278(3):R770–80. 10.1152/ajpregu.2000.278.3.R770. 10.1152/ajpregu.2000.278.3.R770 10712300

[30] Li CH, Pan LH, Li CY, et al. Localization of ANP-synthesizing cells in rat stomach. World J Gastroentero. 2006;12(35):5674–9. 10.3748/wjg.v12.i35.5674. 10.3748/wjg.v12.i35.5674 PMC4088169 17007021

[31] Pang J, Li G, Qian H, et al. Secretory type II cGMP-dependent protein kinase blocks activation of PDGFRβ via Ser254 in gastric cancer cells. Cell Biol Int. 2022;46(5):747–54. 10.1002/cbin.11766. 35066967 10.1002/cbin.11766 PMC9305209

[32] Jonas KC, Melrose T, Thompson IR, et al. Natriuretic peptide activation of extracellular regulated kinase 1/2 (ERK1/2) pathway by particulate guanylyl cyclases in GH3 somatolactotropes. Cell Tissue Res. 2017;369(3):567–78. 10.1007/s00441-017-2624-x. 28451751 10.1007/s00441-017-2624-x PMC5579180

[33] Fu YF, Gui R, Liu J. HER-2-induced PI3K signaling pathway was involved in the pathogenesis of gastric cancer. Cancer Gene Ther. 2015;22(3):145–53. 10.1038/cgt.2014.80. 25613482 10.1038/cgt.2014.80

[34] Sokolova O, Naumann M. Matrix Metalloproteinases in Helicobacter pylori-Associated Gastritis and Gastric Cancer. Int J Mol Sci. 2022;23(3):1883. 10.3390/ijms23031883. 35163805 10.3390/ijms23031883 PMC8836485

[35] Tang Y, Yang H, Yu J, et al. Network pharmacology-based prediction and experimental verification of the involvement of the PI3K/Akt pathway in the anti-thyroid cancer activity of crocin. Arch Biochem Biophys. 2023;743:109643. 10.1016/j.abb.2023.109643. 37211223 10.1016/j.abb.2023.109643

