# Supplementary material for: Renqing Mangjue modulates chronic atrophic gastritis inflammation-cancer transition via the cGMP–PKG/PI3K–AKT pathway
Source: Chin Med. 2026 Jul 14;21:190. doi: 10.1186/s13020-026-01457-2 (PMC13366807; doi:10.1186/s13020-026-01457-2)
Supplement: Supplementary file 1 — Supplementary Material 1 [file 13020_2026_1457_MOESM1_ESM.docx]

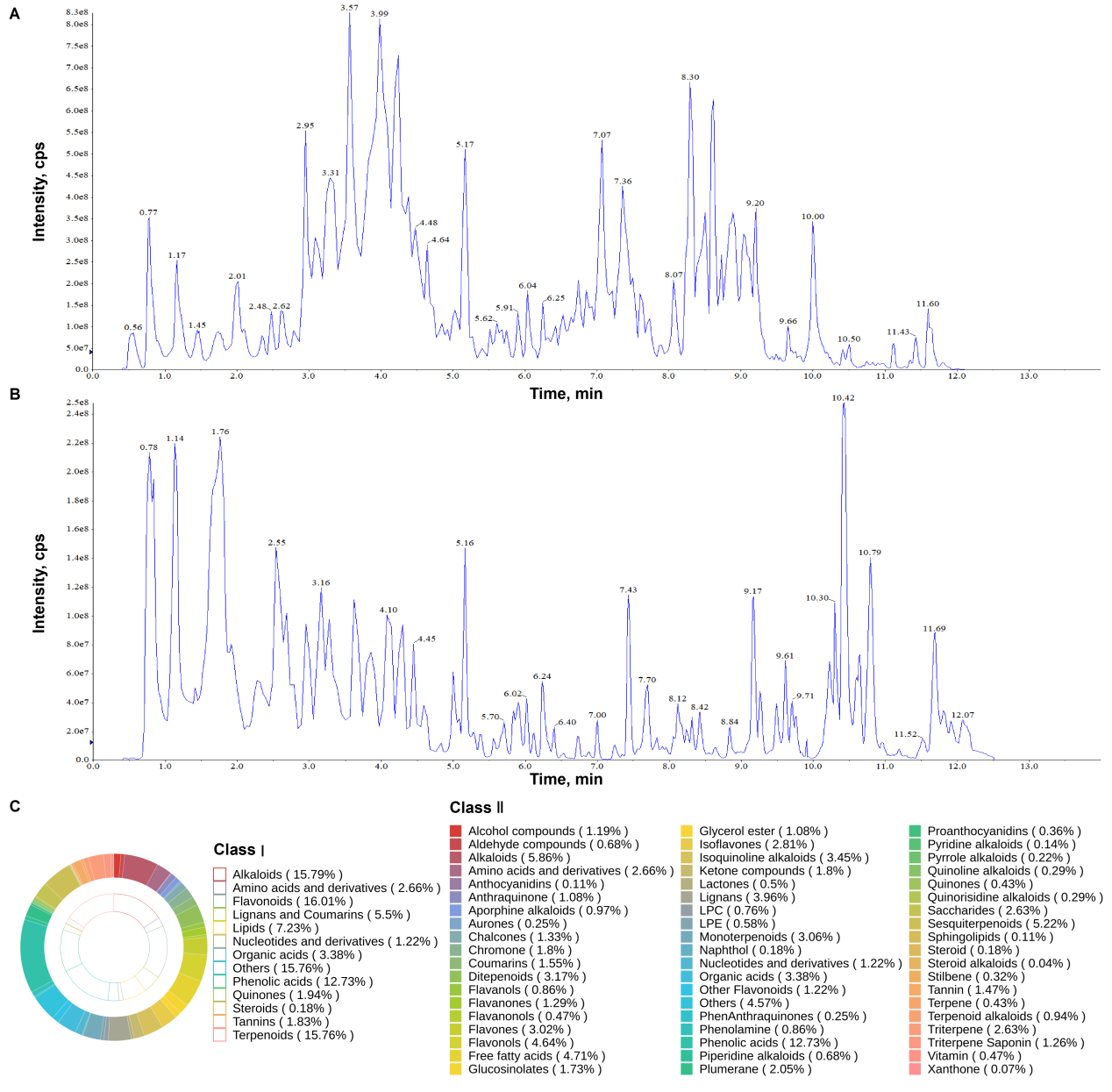


**Supplementary Figure 1. Chemical profiling of RQMJ.** (A) Base peak chromatogram of RQMJ in positive ion mode. (B) Base peak chromatogram of RQMJ in negative ion mode. (C) Classification of all identified metabolites in RQMJ.


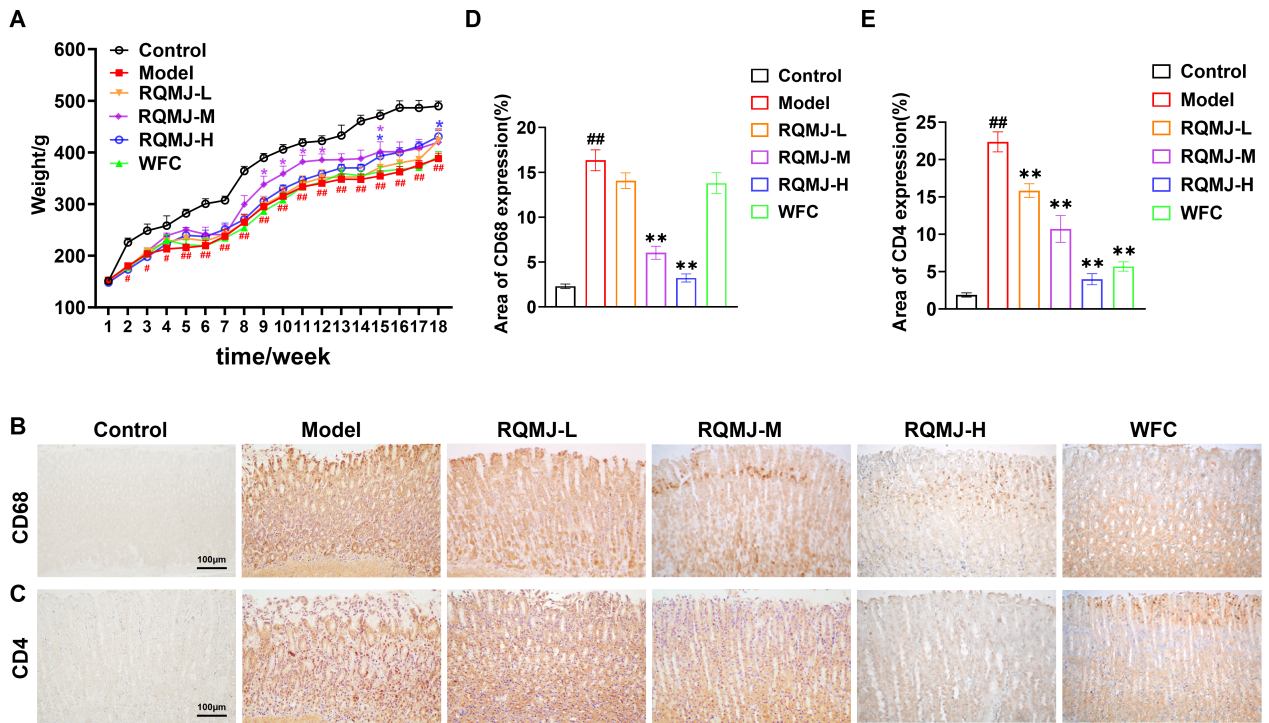


**Supplementary Figure 2. Effects of RQMJ Treatment on Inflammation and Gastric Lesions in CAG Rats.** (A) Body weight changes in control, model, and treatment groups from week 1 to week 18 (*n* = 6). (B–C) Representative IHC staining for CD68 (B) and CD4 (C) in gastric tissues (n = 3; scale bar: 100 μm). (D–E) Quantification of CD68- and CD4-positive staining areas in the gastric mucosa (n = 3). Data are presented as mean ± SEM. ^##^*P* < 0.01, versus Control group; ^**^*P* < 0.01, versus Model group.


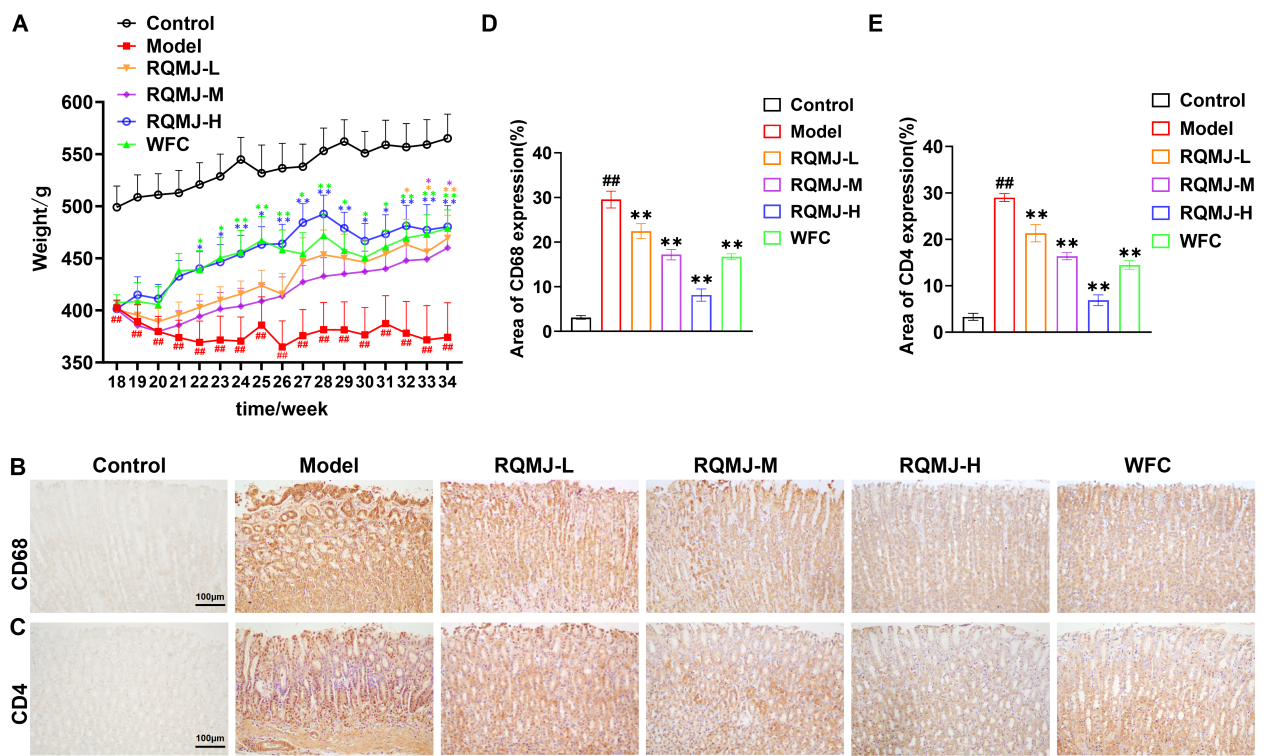


**Supplementary Figure 3. RQMJ intervention in the inflammation–cancer transition of CAG.** (A) Body weight changes from week 18 to week 34 post-modeling in each group (*n* = 6). (B–C) Representative IHC staining for CD68 (B) and CD4 (C) in gastric tissues (n = 3; scale bar: 100 μm). (D–E) Quantification of CD68- and CD4-positive staining areas in the gastric mucosa (n = 3). Data are presented as mean ± SEM. ^##^*P* < 0.01, versus Control group; ^**^*P* < 0.01, versus Model group.


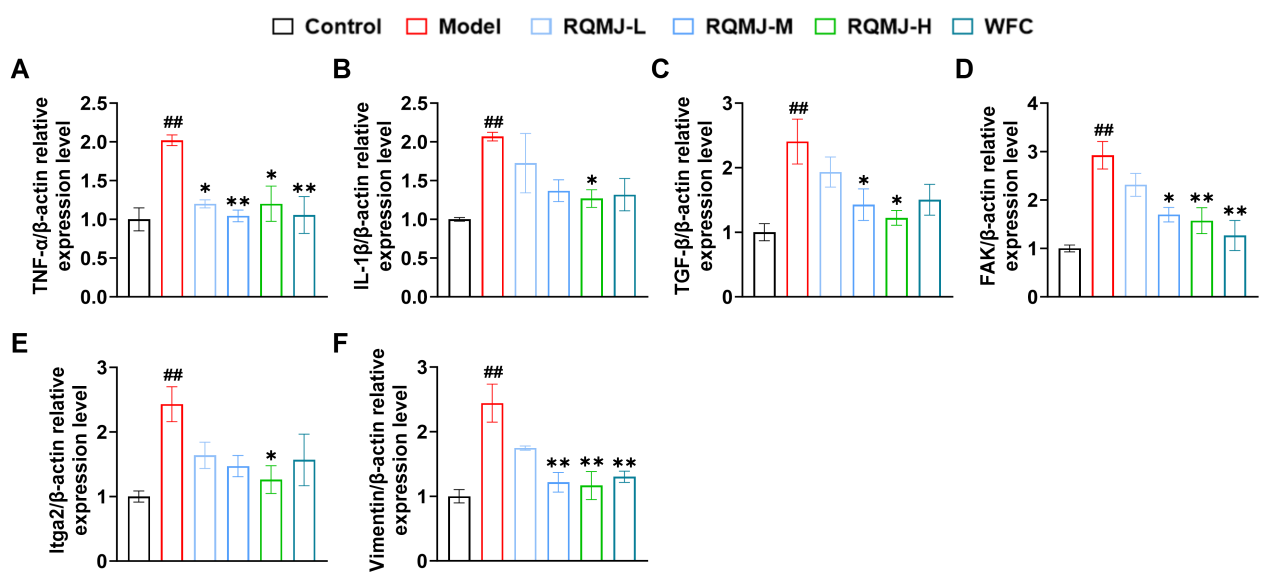


**Supplementary Figure 4. RQMJ inhibits MNNG-induced malignant transformation of gastric mucosal epithelial cells.** (A-F) Densitometric quantification of Western blot bands showing relative expression levels of TNF-α, IL-1β, TGF-β, FAK, Itga2, Vimentin, with β-actin as the internal control (*n* = 3). Data are presented as mean ± SEM. ^#^*P* < 0.05, ^##^*P* < 0.01, versus Control group; ^*^*P* < 0.05, ^**^*P* < 0.01, versus Model group.


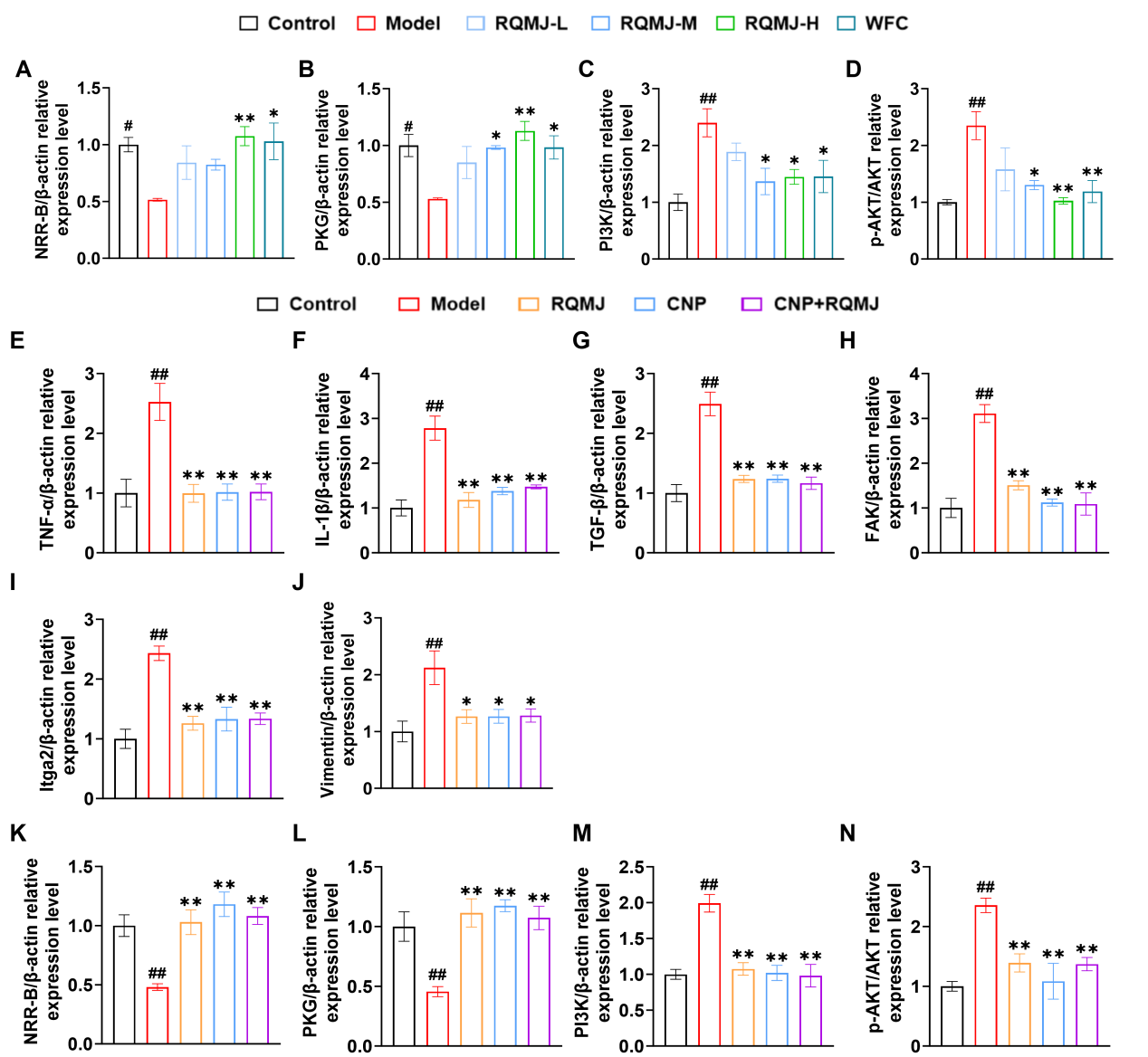


**Supplementary Figure 5. RQMJ inhibits malignant transformation of gastric mucosal epithelial cells via the cGMP–PKG/PI3K–AKT signaling pathway.** (A-D) Densitometric quantification of Western blot bands showing relative expression levels of NPR-B, PKG, PI3K, with β-actin as the internal control (*n* = 3). Quantification of p-AKT expression normalized to total AKT (*n* = 3). (E-N) Densitometric quantification of Western blot bands showing relative expression levels of TNF-α, IL-1β, TGF-β, FAK, Itga2, Vimentin, NPR-B, PKG, PI3K, with β-actin as the internal control (*n* = 3). Quantification of p-AKT expression normalized to total AKT (*n* = 3). Data are presented as mean ± SEM. ^#^*P* < 0.05, ^##^*P* < 0.01, versus Control group; ^*^*P* < 0.05, ^**^*P* < 0.01, versus Model group.
